# Muscarinic acetylcholine receptor-dependent and NMDA receptor-dependent LTP and LTD share the common AMPAR trafficking pathway

**DOI:** 10.1016/j.isci.2023.106133

**Published:** 2023-02-03

**Authors:** Tomonari Sumi, Kouji Harada

**Affiliations:** 1 Research Institute for Interdisciplinary Science, Okayama University, 3-1-1 Tsushima-Naka, Kita-ku, Okayama 700-8530, Japan; 2 Department of Chemistry, Faculty of Science, Okayama University, 3-1-1 Tsushima-Naka, Kita-ku, Okayama 700-8530, Japan; 3 Department of Computer Science and Engineering, Toyohashi University of Technology, Tempaku-cho, Toyohashi 441-8580, Japan; 4 Center for IT-Based Education, Toyohashi University of Technology, Tempaku-cho, Toyohashi, 441-8580, Japan

**Keywords:** Cellular neuroscience, Cellular physiology, Molecular neuroscience

## Abstract

The forebrain cholinergic system promotes higher brain function in part by signaling through the M_1_ muscarinic acetylcholine receptor (mAChR). Long-term potentiation (LTP) and long-term depression (LTD) of excitatory synaptic transmission in the hippocampus are also induced by mAChR. An AMPA receptor (AMPAR) trafficking model for hippocampal neurons has been proposed to simulate N-methyl-D-aspartate receptor (NMDAR)-dependent synaptic plasticity in the early phase. In this study, we demonstrated the validity of the hypothesis that the mAChR-dependent LTP/LTD shares a common AMPAR trafficking pathway associated with NMDAR-dependent LTP/LTD. However, unlike NMDAR, Ca^2+^ influx into the spine cytosol occurs owing to the Ca^2+^ stored inside the ER and is induced via the activation of inositol 1,4,5-trisphosphate (IP3) receptors during M1 mAChR activation. Moreover, the AMPAR trafficking model implies that alterations in LTP and LTD observed in Alzheimer’s disease could be attributed to age-dependent reductions in AMPAR expression levels.

## Highlights

• M_1_ mAChR- and NMDAR-dependent LTP and LTD share the common AMPAR trafficking pathway
• Competition between Ca^2+^-dependent exocytosis and endocytosis regulates LTP and LTD
• A simple reduction in the number of AMPARs weakens LTP and strengthens LTD
• The synaptic dysfunction is correlated with cognitive decline in Alzheimer’s disease

## Graphical abstract

**Figure:**
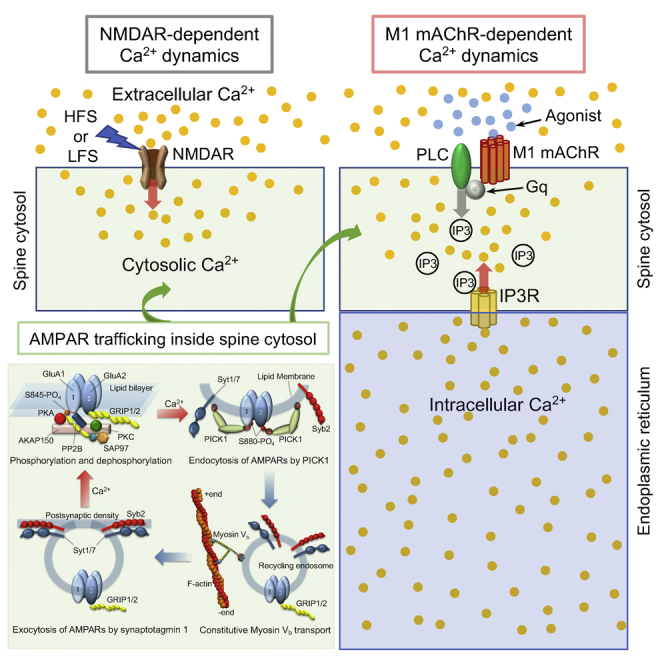

## Introduction

The basal forebrain cholinergic systems innervate the hippocampus and cortex to regulate information processing and higher brain function.^1^^,^^2^ In particular, inhibition of the muscarinic acetylcholine receptors (mAChRs) produces pronounced amnesia, and loss of cholinergic innervation to the hippocampus during Alzheimer’s disease (AD) is thought to contribute to the cognitive deficits observed in AD.^3^^,^^4^^,^^5^ The only available treatment for the cognitive deficits in AD is the use of acetylcholinesterase inhibitors,^6^ which increase the amount of acetylcholine that activates excitatory neurons. In addition, there is increasing interest in the agonists that specifically activate mAChRs for the treatment of both AD^7^^,^^8^ and schizophrenia.^9^ Therefore, it is extremely important to understand how acetylcholine regulates synaptic functions, particularly those involved in learning and memory.

**Figure 1:**
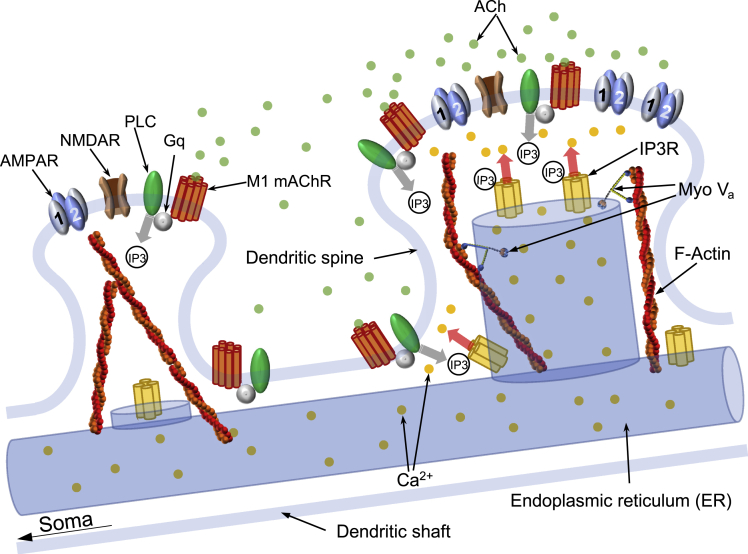
Dendritic spines and ER in hippocampal postsynaptic pyramidal neuron In the larger spine (right-hand side), ER forms a spine apparatus, while in the smaller spine, it does not.^82^ The ER visits the spines by myosin V_a_ transport along F-actin.^82^ Activation of mAChRs with acetylcholine released from presynaptic neurons leads to the coupling of G-proteins (G_q_ or G_11_) to phospholipase C (PLC).^15^ The activated PLC generates diacylglycerol (DAG) and inositol 1,4,5-trisphosphate (IP3) through hydrolysis of the membrane phospholipid phosphatidylinositol-4,5-bisphosphate.^15^ Cytosolic IP3 binds to IP3 receptor (IP3R), a calcium channel, and Ca^2+^ stored inside the ER is released and flows into the spine cytosol.

Activation of mAChRs by agonists induces both long-term potentiation (LTP)^10^ and long-term depression (LTD)^11^^,^^12^^,^^13^^,^^14^ in the hippocampus. However, the molecular mechanisms underlying mAChR-dependent LTP and LTD are poorly understood. M1 mAChR is most abundantly expressed in the hippocampus, cortex, and striatum; localizes to postsynaptic membranes^5^; and signals via the G-proteins G_q_ or G_11_ to activate phospholipase C (PLC).^15^ M1 mAChR-mediated activation of PLC generates diacylglycerol (DAG) and inositol 1,4,5-trisphosphate (IP3) through the hydrolysis of the membrane phospholipid phosphatidylinositol-4,5-bisphosphate.^15^ DAG binds to and stimulates protein kinase C, and IP3 liberates intracellular Ca^2+^ stored inside the ER by binding to the IP3 receptor, an IP3-sensitive calcium channel (see Figure 1).^15^ Therefore, local molecular processes inside dendritic spines, at least downstream of the Ca^2+^ signal, may be in part common between M1 mAChR-dependent and N-methyl-D-aspartate receptor (NMDAR)-dependent synaptic activities, although the sources of Ca^2+^ influx into the spine cytosol for these activities are intracellular Ca^2+^ stored in the ER and extracellular Ca^2+^, respectively.

Extracellular acetylcholine concentrations increase in the hippocampus by as much as 4-fold during a variety of hippocampal-dependent learning tasks,^16^^,^^17^ implying the importance of mAChR signal transmission in learning and memory. Activation of M1 mAChRs in hippocampal pyramidal neurons leads to an increase in cytosolic Ca^2+^ level, which is initiated in apical dendrites and propagates as a wave toward the soma, consequently invading the nucleus.^18^ The increase in nuclear Ca^2+^ levels leads to new gene transcription, for example, the expression of immediate-early genes, such as *c-Fos* and *Arc* RNA.^19^ mAChRs expressed in hippocampal neurons are specifically involved in the modulation of memory.^20^ It is suggested that increased ACh levels are necessary for encoding new spatial contexts, and decreased ACh levels are necessary for retrieving previously learned spatial contexts.^20^ However, the relationship between mAChR-dependent LTP/LTD and new gene expression is not well understood. Here, we propose the hypothesis that M1 mAChR-dependent LTP and LTD share the common α-amino-3-hydroxy-5-methyl-4-isoxazolepropionic acid receptor (AMPAR) trafficking pathway associated with NMDAR-dependent LTP and LTD. In a previous study,^21^ we presented a biochemical network model for AMPAR trafficking in NMDAR-dependent bidirectional synaptic plasticity, where early LTP and LTD in adult rat hippocampal pyramidal neurons were reproduced without considering protein synthesis.^22^^,^^23^^,^^24^ In the present study, we show that the molecular mechanism of AMPAR trafficking, which is common with NMDAR-dependent bidirectional synaptic plasticity downstream of Ca^2+^ signaling, causes both M1 mAChR-dependent LTP and LTD. This finding provides further evidence supporting the validity of a unified molecular mechanism of Ca^2+^-dependent AMPAR trafficking in hippocampal bidirectional synaptic plasticity.

## Method

### Endoplasmic reticulum model of Ca^2+^ dynamics

**Figure 2:**
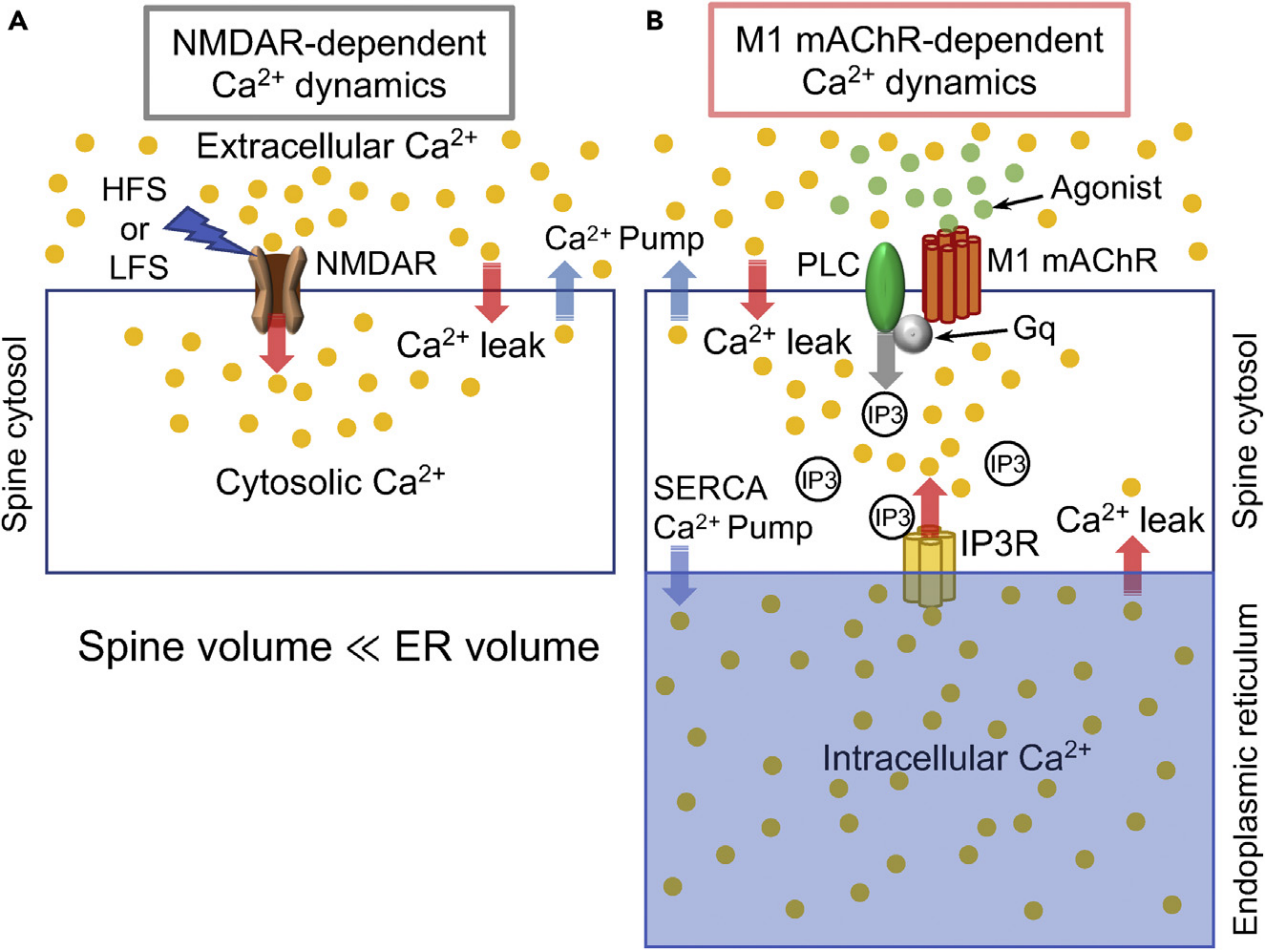
Model of Ca^2+^ dynamics for NMDAR-dependent and M1 mAChR-dependent LTP and LTD in hippocampal neurons (A) Model of Ca^2+^ dynamics for NMDAR-dependent LTP and LTD simulations.^21^ The influx of extracellular Ca^2+^ into spine cytosol via NMDARs during high-frequency stimulation (HFS) and low-frequency stimulation (LFS) was used as the input for LTP and LTD simulations, respectively, although no NMDARs were explicitly included in the model. (B) ER model of Ca^2+^ dynamics for M1 mAChR-dependent LTP and LTD simulations. The influx of intracellular Ca^2+^ from the ER into the spine cytosol was mediated by IP3 receptors upon IP3 binding, where the flux of IP3 production induced by M1 mAChR signal was used as the input of the mLTP and mLTD simulations; no M1 mAChRs were explicitly included in the model (see STAR Methods).

In a previous study on NMDAR-dependent LTP and LTD,^21^ the influx of extracellular Ca^2+^ into the spine cytosol via the NMDAR ion channel (Figure 2A) during high-frequency stimulation (HFS) at 100 Hz for 1 s and low-frequency stimulation (LFS) with 700-900 pulses at 1 Hz was modeled using a single Gaussian (or sigmoidal) function and multiple Gaussian (or sigmoidal) functions, which were employed as the input for the simulations. In these models, Ca^2+^-leak influx and Ca^2+^-pump outflux were also incorporated. In contrast, in the present study on M1 mAChR-dependent LTP (mLTP) and LTD (mLTD), in addition to the same Ca^2+^ leak and Ca^2+^-pump fluxes, Ca^2+^-pump flux from the cytosol to the ER, which is caused by sarcoendoplasmic reticulum calcium ATPase (SERCA),^25^ as well as Ca^2+^-leak flux from the ER to the cytosol, were taken into consideration (Figure 2B). Furthermore, Ca^2+^ influx from the ER via the IP3 receptor, an IP3-sensitive ion channel, was incorporated (Figure 2B).^26^ This Ca^2+^ influx is triggered by IP3 binding to the IP3 receptor, which depends on the concentration of IP3. IP3 molecules are generated by PLC when PLC is activated by M1 mAChR signaling, which is induced upon binding of the agonist to M1 mAChR and transmitted to PLC via G_q_ or G_11_.^15^ In the present study, the flux of IP3 production mediated by PLC activation was used as the input for simulations of mLTP and mLTD. A mathematical model of Ca^2+^ dynamics arising from the ER is provided in STAR Methods.

### Summary of the network model for α-amino-3-hydroxy-5-methyl-4-isoxazolepropionic acid receptor trafficking

**Figure 3:**
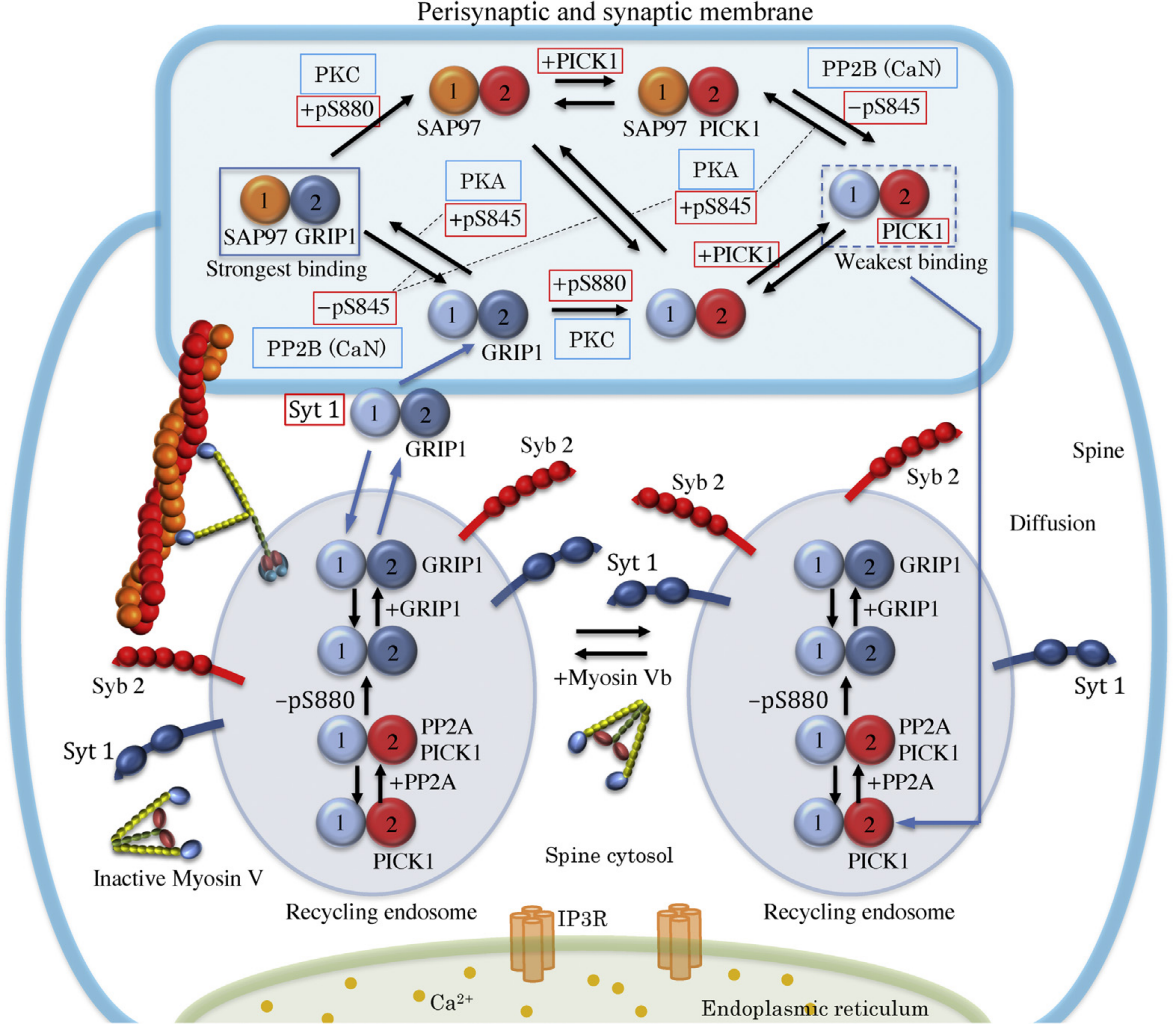
The network model for AMPAR trafficking that mediates hippocampal mLTP and mLTD For simplification, the AMPAR is schematically depicted as two particles corresponding to the GluA1 and GluA2 subunits. However, in the network model, it is treated as a heterotetramer consisting of two GluA1 and two GluA2 subunits. The reaction network for the phosphorylation/dephosphorylation dynamics of GluA1 and GluA2 is also displayed schematically. The recycling endosomes containing AMPAR are actively transported by myosin V_b_ toward the perisynaptic/synaptic membrane. The lateral diffusion relocation of AMPAR is assumed to occur during the phosphorylation and dephosphorylation of AMPAR at the synaptic membrane, in addition to the local diffusional relocation movement of the exocytic AMPAR from the perisynaptic to the synaptic membrane. The phosphorylation state of GluA1 and GluA2 regulates the localization of the AMPARs at the synaptic membrane via interactions with various AMPAR-interacting proteins (SAP97, GRIP1, PICK1).^32^

In a previous study, we proposed a network model for bidirectional hippocampal synaptic plasticity, including: (1) Ca^2+^ dynamics, (2) the phosphorylation/dephosphorylation dynamics of the tetrameric AMPAR ion channel subtype GluA1/A2, (3) the endocytosis/exocytosis dynamics of AMPARs, mediated by the Ca^2+^-sensors protein interacting with C-kinase 1 (PICK1) and synaptotagmin 1 (Syt1), and (4) the active transport of recycling endosomes containing AMPARs by molecular motor myosin V_b_ toward the perisynaptic/synaptic membrane (Figure 3 and STAR Methods). In hippocampal pyramidal neurons, the GluA1/A2 heterotetramer is the most dominant AMPAR subtype, followed by the GluA2/A3 heterotetramer^27^; therefore, we incorporated the GluA1/A2 heterotetramer in the model of AMPAR trafficking.

Phosphorylation and dephosphorylation of GluA1/A2 heterotetramers in postsynaptic membranes are regulated by the A-kinase anchoring protein 150 (AKAP150) signaling complex^28^ along with protein kinase A (PKA), protein kinase C (PKC),^29^ and protein phosphatase 2 B (PP2B, also known as calcineurin [CaN]) (Figure 3).^30^ If the serine-845 site (S845) of GluA1 is phosphorylated by cAMP-dependent PKA,^31^ then the synapse-associated protein of 97 kDa (SAP97) binds to GluA1 (Figure 3).^32^^,^^33^ Thus, GluA1 is tethered to AKAP150 via SAP97, because AKAP150 has a MAGUK binding domain that interacts with SAP97 ^33^, and SAP97 has PDZ domains that interact with the phosphorylated S845 site of GluA1.^32^ In contrast, if S845 of GluA1 is dephosphorylated by Ca^2+^-dependent PP2B,^30^ SAP97 dissociates from GluA1 (Figure 3). If S880 of GluA2 is phosphorylated by Ca^2+^-dependent PKC,^34^^,^^35^ then glutamate receptor-interacting protein 1 (GRIP1), which interacts with the dephosphorylated S880 site of GluA2 via the PDZ domains of GRIP1,^32^^,^^36^ dissociates from GluA2 (Figure 3). As a result, the Ca^2+^-sensor protein PICK1^34^^,^^37^^,^^38^ binds to S880 of GluA2 (Figure 3).

PICK1 is activated by an increase in the cytosolic Ca^2+^ concentration and mediates Ca^2+^-dependent endocytosis, together with clathrin and dynamin (Figure 3).^39^ Functions of these collaborating proteins that are not explicitly incorporated here are considered in the AMPAR trafficking model through effective rate constants for PICK1-dependent endocytosis. The newly generated endocytic vesicles containing AMPARs diffuse in the spine cytosol (Figure 3). During this process, it is assumed that S880 of GluA2 in the recycling endosomes is dephosphorylated by PP2A,^40^ PICK1 dissociates from GluA2, and GRIP1 binds to GluA2 (Figure 3).^32^^,^^36^ In parallel, myosin V_b_ binds to the recycling endosome via Rab11 and transports it toward the perisynaptic/synaptic membrane (Figure 3).^41^^,^^42^^,^^43^^,^^44^ In general, the normal lateral diffusion of AMPARs along plasma membrane surfaces is isotropic^45^ and is therefore thought to be ineffective for long-range directional transport of AMPARs. Instead, myosin V_b_-mediated transport of recycling endosomes, which is driven by molecular motors that consume ATP as fuel,^46^ is responsible for long-range directional transport of AMPARs.

Recycling endosome exocytosis is mediated by Ca^2+^-sensor synaptic vesicle proteins, including Syt1, synaptotagmin 7 (Syt7), synaptobrevin-2/VAMP2, and complexin.^47^^,^^48^^,^^49^^,^^50^^,^^51^^,^^52^ In the same manner as PICK1-dependent endocytosis, functions of these collaborating proteins that are not explicitly incorporated are considered in the AMPAR trafficking model through effective rate constants for Syt1-dependent endocytosis. As a result, the AMPARs are incorporated into the perisynaptic/synaptic membrane (Figure 3). In fact, exocytosis of GluA1 in dendrites and dendritic spines, including perisynaptic/synaptic membranes, has been demonstrated using SEP-GluA1 imaging.^44^ Furthermore, localization of Syt1 at the perisynaptic/synaptic membranes has been observed in hippocampal postsynaptic neurons.^53^ These experimental observations provide convincing evidence supporting the validity of the model for AMPAR trafficking. The AMPARs incorporated into the perisynaptic membrane are relocated into the synaptic membrane via a local diffusional movement, which takes at most 2 min, as shown by the observation of the duration of short-term potentiation.^54^ In the present study, the ∼2-min delay of AMPAR incorporation into the synaptic membrane after exocytosis at the perisynaptic membrane was taken into consideration via effective rates for Syt1-dependent exocytosis. The diffusional relocation dynamics of AMPARs at the synaptic membrane play a crucial role in their interaction with AKAP150, thereby stabilizing AMPARs at the synaptic membrane by tethering to AKAP150 via SAP97 and GRIP1.

### Simulations

A network model consisting of ordinary differential equations (ODEs) was applied to mLTP and mLTD induction in adult hippocampal neurons. The flux of IP3 molecules generated by PLC activated by M1 mAChR-dependent signals was employed as the input for the mLTP and mLTD simulations. Details of the input flux of IP3 are provided in STAR Methods. Steady-state concentrations were used as initial conditions for the simulations. The ODEs were solved for 5400 s using a COPASI biochemical system simulator (ver. 4.37).^55^

## Results

### Ca^2+^ influx into the spine cytosol from endoplasmic reticulum induces muscarinic acetylcholine receptor-dependent long-term potentiation and muscarinic acetylcholine receptor-dependent long-term depression

**Figure 4:**
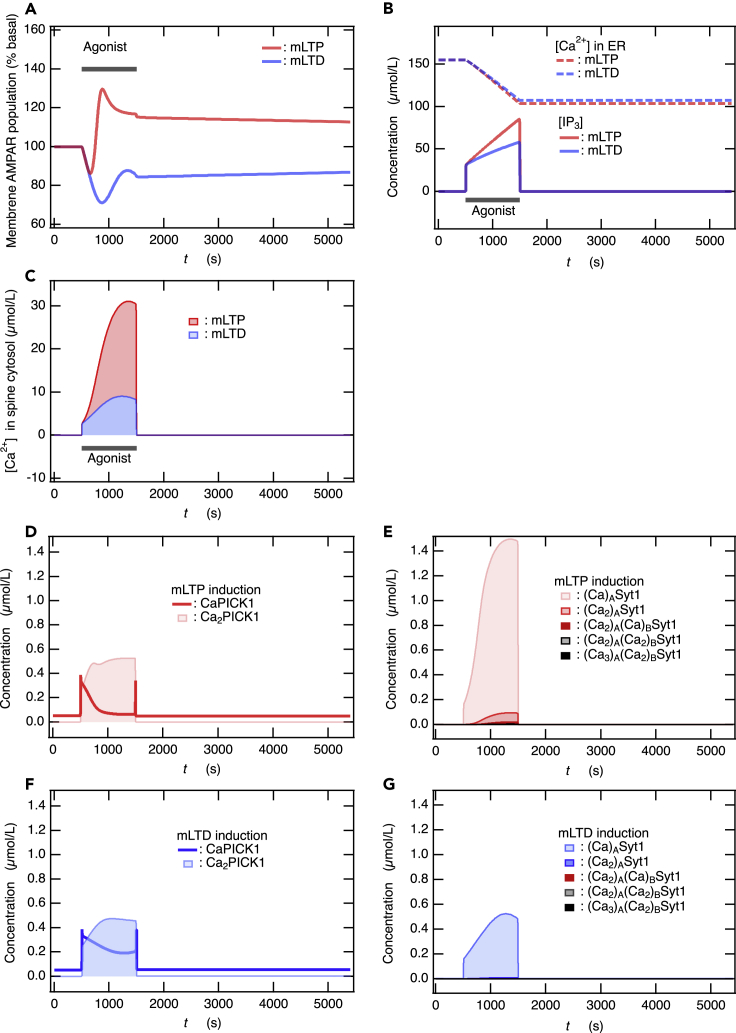
M1 mAChR-dependent hippocampal LTP and LTD are induced by the activation of the Ca^2+^ sensors Syt1 and PICK1 in response to Ca^2+^ influx into the spine cytosol from ER (A) Time course of the membrane AMPAR population, indicating the induction of mLTP and mLTD. Here, 100% represents the basal AMPAR population at the membrane. (B) Time course of [Ca^2+^] inside the ER and of [IP3], during the mLTP and mLTD induction. (C) The transient rise in [Ca^2+^] during the mLTP and mLTD induction. (D-G) The concentrations of Ca^2+^-binding species of PICK1 (D and F) and Syt1 (E and G) as a function of time (*t*) during the induction of mLTP (D and E) and mLTD (F and G). In (G), the concentration of all species with Ca^2+^ binding to C_2B_ domain in Syt1 is extremely low to be detected at this scale, indicating that Syt1 is not sufficiently activated. Therefore, a small amount of Syt1-mediated exocytosis occurs during the mLTD.

Typical and characteristic forms of mLTP and mLTD have been observed experimentally. For instance, it has been observed that the application of 50 μM carbachol, which is a cholinergic receptor agonist, for 10 min results in mLTD, and the synaptic transmission decreases down to ∼20% compared to basal levels.^11^^,^^12^^,^^13^^,^^14^ However, thereafter, it increases to be between 60% and 80%, resulting in mLTD induction.^11^^,^^12^^,^^13^^,^^14^ Our network model reproduces the characteristic behavior that is qualitatively consistent with the experimentally observed mLTD induction mentioned above (Figure 4A) a flux of IP3 production by PLC is provided so that IP3 concentration rises (Figure 4B). On the contrary, it has been observed experimentally that the application of brief, localized, acetylcholine puffs at 1 mM for 300 ms decreases synaptic transmission down to ∼40% and subsequently increases up to ∼200%, resulting in mLTP induction.^10^ The duration of applying acetylcholine puffs is much shorter than the application of carbachol for mLTD. However, the concentration of acetylcholine is higher than that of carbachol, and acetylcholine is not washed out.^10^ Thus, acetylcholine is expected to be kept at high concentrations. On the other hand, in the induction of mLTD, carbachol is often washed out after its application.^12^^,^^13^ Therefore, the activation of M1 mAChR on mLTP induction would be stronger than that on mLTD and can be continued for a duration sufficiently longer than the 300 ms duration while applying acetylcholine puffs. Hereafter, we use square brackets to refer to the concentrations. When the increase in [IP3] is higher than that for mLTD (Figure 4B) so that the increase in cytosolic [Ca^2+^] is much higher than that for mLTD (Figure 4C), our network model reproduces the time course of the change in membrane AMPAR population; this result is consistent with the experimentally observed characteristic behavior of mLTP induction. Specifically, the synaptic transmission or membrane AMPAR population drops down once initially but then increases and consequently becomes higher than the basal level (Figure 4A).^10^ In addition, we found that the population of membrane AMPARs monotonically varied from mLTD to mLTP induction when the increase in [IP3] and the resulting increase in cytosolic [Ca^2+^] gradually became higher (see Figure S1 in Supplemental Information (SI)). In the mLTP simulations, for comparison, the activation of M1 mAChR by the application of acetylcholine puff for 300 ms was assumed to last for the same duration, aligned with the mLTD induction. However, we confirmed that qualitatively similar mLTP induction was observed even when the duration of activation was halved (see Figure S2 in the SI). We observed that a further decrease in the duration of activation reduced the initial decrease in membrane AMPAR population (Figure S3 in the SI).

The initial decrease in EPSC amplitude during the induction of mLTP is interpreted to be due to presynaptic mAChR-mediated inhibition of N-type Ca^2+^ channels.^10^ The inhibition effect is not considered in our network model, whereas the presented simulation result indicates a possibility that the initial decrease in the observed EPSC amplitude is due to a decrease in postsynaptic AMPARs because of the initial dominance of endocytosis. A slight initial decrease in AMPARs can also be observed during the rapid induction of NMDAR-dependent LTP,^21^ although it may be hard to experimentally detect it. This reflects a generality of the initial dominance of Ca^2+^-mediated endocytosis in our AMPAR trafficking network model.

The network model exhibits a slow reduction in the amplitude of mLTP and mLTD toward the basal state, which has been experimentally observed, at least for mLTD.^12^^,^^13^^,^^14^ The mLTD and mLTP yielded by the network model, which consists of AMPAR trafficking without protein synthesis, would correspond to the early phases of LTD and LTP. The relaxation of mLTD and mLTP toward the basal state is mediated by constitutive endocytosis and exocytosis of AMPAR, reflecting that the constitutive flux of the recycling endosome occurs under basal conditions in the simulations.

### Both PICK1 and Syt1 are activated during muscarinic acetylcholine receptor-dependent long-term potentiation and muscarinic acetylcholine receptor-dependent long-term depression induction

In the network model for AMPAR trafficking that we developed for the study of NMDAR-dependent LTP and LTD,^21^ the Ca^2+^ sensors Syt1 and PICK1 play a dominant role in the exocytosis and endocytosis of AMPARs that regulate LTP and LTD induction, respectively. Herein, the biochemical features and synaptic plasticity-related functions of Syt1 and PICK1 are briefly reviewed. Syt1 has two Ca^2+^-binding domains, C_2A_ and C_2B_, which bind three and two Ca^2+^ ions, respectively, and directly interact with the recycling endosome and postsynaptic plasma membrane.^49^ PICK1 has two Ca^2+^-binding domains, one each at the N-terminus and the C-terminus, which interact with the phosphorylated S880 of GluA2 and the postsynaptic plasma membrane.^34^^,^^37^ It has been demonstrated that Ca^2+^-binding site mutations in Syt1 in both the C_2A_ and C_2B_ domains block hippocampal LTP.^51^ Furthermore, LTD induced by LFS is modestly affected in juvenile PICK1-knockout (KO) mice, whereas the LTD is significantly reduced in adult PICK1-KO mice.^56^ Therefore, although many factors other than PICK1 work in Ca^2+^-dependent endocytosis of AMPARs during LTD in the hippocampal neurons of juvenile rodents, PICK1 plays a dominant role in the regulation of Ca^2+^-dependent endocytosis of AMPARs in adult rodents, together with other proteins, including clathrin and dynamin.^39^

During the induction of mLTP, the concentrations of Ca^2+^-binding species of Syt1, i.e., [(Ca)_A_Syt1], [(Ca_2_)_A_Syt1], and [(Ca_2_)_A_(Ca)_B_Syt1], gradually increased; however, [(Ca_2_)_A_(Ca_2_)_B_Syt1] and [(Ca_3_)_A_(Ca_2_)_B_Syt1] did not increase significantly, as shown in Figure 4E. [Ca_2_PICK1] increased with a slight delay following a rapid increase in [CaPICK1] (Figure 4D). Therefore, both Syt1 and PICK1 were activated during mLTP. Nevertheless, mLTP is finally induced because Syt1-mediated exocytosis later overcomes PICK1-mediated endocytosis, although the population of membrane AMPARs is initially reduced. In contrast, during mLTD, no increase was observed in the concentration of all the species that lead to Ca^2+^ binding with the C_2B_ domain in Syt1 (Figure 4G), whereas [Ca_2_PICK1] and [CaPICK1] increased in relation to the increase in [Ca^2+^] (Figure 4F). As a result, PICK1-mediated endocytosis overcomes Syt1-mediated exocytosis, causing mLTD induction.

### Competition between endocytosis and exocytosis of α-amino-3-hydroxy-5-methyl-4-isoxazolepropionic acid receptors regulates the induction of muscarinic acetylcholine receptor-dependent long-term potentiation and muscarinic acetylcholine receptor-dependent long-term depression

**Figure 5:**
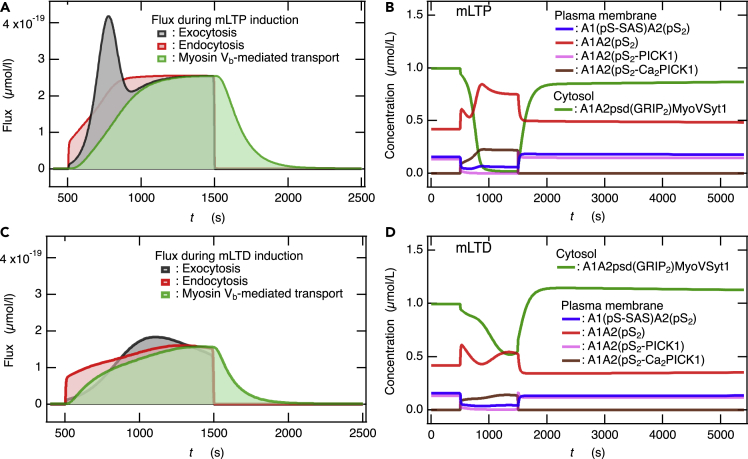
Competition between exocytosis and endocytosis of AMPARs yields mLTP and mLTD (A and C) Total fluxes of exocytosis, endocytosis, and myosin V_b_-mediated transport of recycling endosomes during (A) mLTP stimulation and (C) mLTD stimulation. (B and D) The time course of concentrations for predominant membrane components during (B) mLTP and (D) mLTD.

As predicted from the time course of the change in membrane AMPAR population during mLTP stimulation (Figure 4A), at the early stage of mLTP stimulation, the endocytosis flux was larger than the exocytosis flux (Figure 5A), resulting in an initial reduction in the membrane AMPAR population. However, following this, the exocytosis flux became larger than the endocytosis flux (Figure 5A), and consequently, mLTP was established (Figure 4A). However, during mLTD stimulation, the endocytosis flux was larger than the exocytosis flux (Figure 5C) until the membrane AMPAR population was sufficiently decreased (Figure 4A). However, after reaching a minimum, because the exocytosis flux slightly overcame the endocytosis flux (Figure 5C), the membrane AMPAR population increased to be larger than 80% (Figure 4A). As observed in NMDAR-dependent LTP and LTD,^21^ the maximum endocytic flux in response to mLTD stimulation (∼1.6 × 10^−19^ μmol/s, Figure 5C) is smaller than the maximum endocytic flux in response to mLTP stimulation (∼2.6 × 10^−19^ μmol/s, Figure 5A). This result can be attributed to the fact that the [Ca^2+^] during mLTP stimulation is significantly higher than that during mLTD stimulation (Figure 4C). Nevertheless, mLTD is induced by a smaller endocytic flux than that during mLTP stimulation because, during mLTD, the exocytic flux is sufficiently smaller than the endocytic flux for a while, especially at the early stage of stimulation (Figure 5C). Likewise, mLTP is induced even though the endocytic flux is larger than that during mLTD stimulation because, during mLTP stimulation, the maximum exocytic flux is sufficiently higher than the endocytic flux.

It is remarkable that an increase in the cytosolic AMPAR levels internalized by the endosome during LTP induction could be observed experimentally,^57^ which is convincing evidence that supports the competition mechanism of bidirectional synaptic plasticity. Endocytic vesicles containing AMPARs generated upon the induction of mLTP and mLTD undergo diffusion in the spine cytosol resulting in a recycling endosome, which then binds to molecular motor myosin V_b_ via Rab11 and is actively transported toward the perisynaptic/synaptic membrane.^41^^,^^42^^,^^43^ The transportation flux of the recycling endosomes by myosin V_b_ was observed following the endocytic flux during the induction of mLTP and mLTD and continued for ∼8 min from the end of the agonist stimulation (Figures 5A and 5C). These simulation results are consistent with the experimental observations where the cooperative movements of the recycling endosomes and myosin V_b_ molecules start a few minutes later than LTP stimulation and continue for several minutes following the induction of LTP.^43^

### Recycling endosomes transported by myosin V_b_ are bound to the intracellular surface of perisynaptic/synaptic membranes for the next round of exocytosis as a readily releasable pool of α-amino-3-hydroxy-5-methyl-4-isoxazolepropionic acid receptor

In our network model, the myosin V_b_-mediated active transport was assumed to occur in a Ca^2+^-independent manner, based on experimental observations.^58^^,^^59^ Thus, the recycling endosomes in the spine cytosol are constitutively transported by the myosin V_b_ toward the perisynaptic and synaptic membranes. Consequently, the recycling endosomes are expected to localize on the membrane surface under basal conditions. In fact, the most dominant components in the cytosol under basal conditions (*t* = 0 s) are recycling endosomes bound to the perisynaptic/synaptic membrane surface, namely A1A2psd(GRIP_2_)MyoVSyt1 (Figures 5B and 5D).

A decrease in [A1A2psd(GRIP_2_)MyoVSyt1] occurred after mLTP stimulation, until it was depleted by Syt1-mediated exocytosis (Figure 5B). In fact, the Syt1-mediated exocytic flux gradually increased, reached a peak, and then decreased (Figure 5A) because of the depletion of [A1A2psd(GRIP_2_)MyoVSyt1]. The decrease in the membrane AMPAR population following the peak (Figure 4A) was attributed to the PICK1-mediated endocytic flux overcoming the Syt1-mediated exocytic flux (Figure 5A). The increase in [A1A2psd(GRIP_2_)MyoVSyt1] following the depletion (Figure 5B) is attributed to both the suspension of Syt1-dependent exocytosis and myosin V_b_-mediated transport of the endosomes newly internalized into the spine cytosol through endocytosis (Figure 5A). Likewise, endocytic vesicles internalized during mLTD induction are also transported by myosin V_b_ and are more abundant on the membrane surface than at the basal level (Figure 5D); thus, they play an important role in exocytosis due to subsequent LTP/mLTP stimulation.

### Validity of the network model in M1 muscarinic acetylcholine receptor-dependent synaptic plasticity

**Figure 6:**
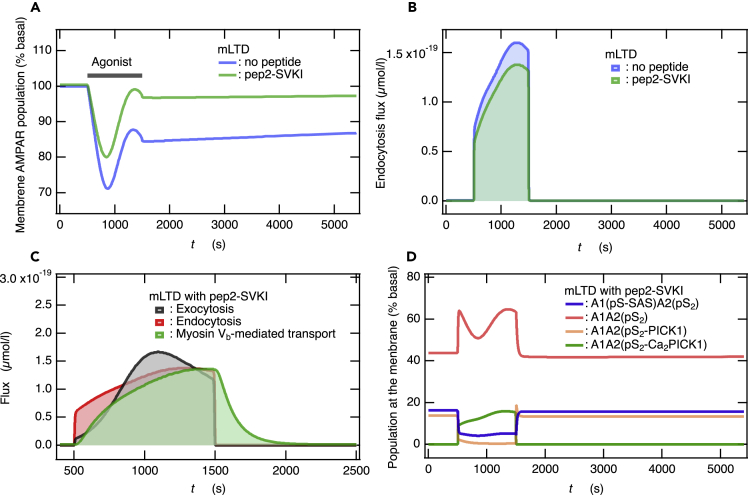
The interference of interactions between GluA2 and PICK1 by pep2-SVKI impairs mLTD (A and B) Time courses of the membrane AMPAR population (A) and the total endocytosis flux (B) due to the mLTD stimulation with and without pep2-SVKI, which interferes with the interactions between GluA2 and PICK1. (C) The fluxes of exocytosis, endocytosis, and myosin V_b_-mediated transport caused by the mLTD stimulation under the inhibitory action of pep2-SVKI. (D) The time course of concentrations for the main components at the membrane during mLTD stimulation under the inhibitory action of pep2-SVKI.

The effects of two peptides, pep2-EVKI (YNVYGIEEVKI) and pep2-SVKI (YNVYGIESVKI), on the induction of hippocampal mLTD have been examined experimentally; pep2-EVKI had no significant effect, while the pep2-SVKI impaired mLTD.^13^ Since pep2-SVKI binds to both GRIP1 and PICK1 *in vitro*, whereas pep2-EVKI binds to PICK1 only,^60^ it has been suggested that GRIP1 rather than PICK1 is involved in mLTD.^13^ However, excluding PICK1 from mLTD, referring only to the observations with pep2-EVKI, may lead to incorrect conclusions. Indeed, a peptide inhibitor pep2-AVKI, which disrupts the binding of GluA2 to PICK1, blocks the induction of mLTD.^61^ In the present study, to confirm the validity of the network model for M1 mAChR-dependent hippocampal synaptic plasticity, we applied the model to the inhibition of mLTD by pep2-SVKI.^60^ The peptide inhibitor pep2-SVKI binds to PICK1, and inhibits PICK1 function, thus suppressing PICK1-dependent endocytosis. We supposed that a dosage of pep2-SVKI decreased PICK1 function by 20%, and then examined the case that all parameters of endocytosis mediated by Ca^2+^-binding PICK1 were reduced to 80% (Table S3 in the SI table). Our network model reproduced the impairment of mLTD experimentally observed by the inhibition of PICK1 function by pep2-SVKI^13^ if PICK1-mediated endocytosis flux was reduced to the extent shown in Figure 6B. Specifically, the membrane AMPAR population decreased at the beginning of mLTD stimulation, whereas later it almost returned to the basal level (Figure 6A). Consequently, the concentrations of the main components of the membrane after mLTD stimulation did not change from the basal state before stimulation (Figure 6D). The re-increase following the first decrease after agonist application in the membrane AMPAR population, which is consistent with the experimentally observed variation in synaptic transmission, is attributed to Syt1-mediated exocytic flux overcoming the endocytic flux (Figure 6C). Such a re-increase in synaptic transmission experimentally observed with pep2-SVKI as well as without peptide shows the occurrence of exocytosis flux caused by mLTD stimulation. These observations again provide convincing evidence supporting the unified mechanism of LTP and LTD induction proposed by us based on the competition between exocytosis and endocytosis.^21^

The inhibitory effects of PICK1 by pep2-SVKI were also examined for the NMDAR-dependent induction of LTD and LTP. The induction of LTD was impaired in a similar manner to that of mLTD, and the induction of LTP was not affected at all (see Figure S4), which was not expected. These simulation results are consistent with experimental observations for adult PICK1-KO mice,^56^ supporting the validity of the network model.

**Figure 7:**
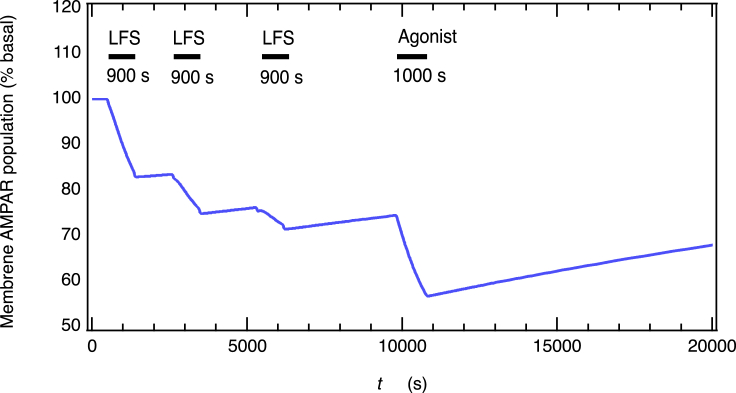
mLTD induction is not occluded even after NMDAR-dependent inductions of LTD are saturated The obvious induction of mLTP following saturated inductions of LTD seems to be consistent with the experimental observation.^12^ Simulation details are provided in Tables S5 and S6.

As another demonstration to confirm the validity of the network model, the so-called occlusion experiment of mLTD induction was examined by utilizing the saturated inductions of NMDAR-dependent LTD (Figure 7). First, NMDAR-dependent inductions of LTD were saturated by three episodes of LFS, followed by mLTD stimulation, (which is the same as that used in Figure 4), applied between 9800 s and 10,800 s (Tables S5 and S6 in the SI table). Unexpectedly, the subsequent induction of mLTD was not occluded by the preceding saturated inductions of LFS-mediated LTD, even though the mLTD induction shares a common AMPAR trafficking mechanism with the NMDAR-dependent LTD (Figure 7). The induction of mLTP following saturated inductions of LTD simulated by the AMPAR trafficking network model seems to be consistent with the experimental observation.^12^ These observations indicate whether the induction of mLTD is occluded or not by the saturation of the preceding LTD induction and are not necessarily dependent on whether the mAChR-dependent LTD and NMDAR-dependent LTD share a common AMPAR trafficking mechanism.

## Discussion

### M1 muscarinic acetylcholine receptor-dependent and N-methyl-D-aspartate receptor-dependent synaptic plasticity share a common α-amino-3-hydroxy-5-methyl-4-isoxazolepropionic acid receptor trafficking mechanism that does not require gene expression, at least in the early stages of long-term potentiation and long-term depression

In our previous study,^21^ we presented a biochemical network model for AMPAR trafficking in NMDAR-dependent bidirectional synaptic plasticity and demonstrated that early LTP and LTD in adult rat hippocampal pyramidal neurons without protein synthesis were reproduced, which was consistent with previous experiments.^22^^,^^23^^,^^24^ In the model, HFS- and LFS-induced Ca^2+^ influx into the spine cytosol through NMDAR ion channels was introduced as the input for LTP and LTD simulations. In the present study, to verify the hypothesis that M1 mAChR-dependent induction of LTP and LTD shares the common AMPAR trafficking pathway with NMDAR-dependent synaptic plasticity, an M1 mAChR-dependent ER model for Ca^2+^ dynamics was developed and incorporated into the AMPAR trafficking network model.^21^ It has been observed that the activation of M1 mAChR raises nuclear calcium levels,^18^ which should be attributed to calcium release from the ER via the IP3 receptor. The release of calcium from the ER and the consequent rise in nuclear calcium levels have been shown to be effective in initiating gene transcription via phosphorylation of cAMP response element-binding protein (CREB).^62^^,^^63^^,^^64^ Long-term synaptic changes associated with memory and learning require the expression of genes such as *C-FOS* and brain-derived neurotrophic factor (*BDNF*), which are regulated by CREB. However, protein synthesis due to gene expression was not considered in the present model. Nevertheless, the incorporated AMPAR trafficking pathway successfully reproduced the characteristic induction forms of LTP and LTD mediated by the M1 mAChR and NMDAR. Therefore, it can be concluded that the M1 mAChR-dependent induction of LTP and LTD shares the common AMPAR trafficking pathway with NMDAR-dependent synaptic plasticity, and new gene expression is not necessary, at least in the early stages of LTP and LTD.

### Synaptic dysfunction and the resulting synaptic loss are correlated with cognitive decline in Alzheimer’s disease

Accumulating evidence has demonstrated that cognitive decline in AD is caused by amyloid β (Aβ), which induces synaptic dysfunction and loss via tau protein.^65^ Indeed, alterations in hippocampal LTP and LTD have been observed as a phenotype of synaptic dysfunction in animal models of AD.^66^ Furthermore, abnormalities in the expression profile of immediate-early genes, such as *CREB*, have been found in the brains from patients with AD ^67^ as well as AD mouse models.^68^^,^^69^^,^^70^ Degeneration of basal forebrain cholinergic neurons is one of the neuropathological features observed in the brains of patients with AD,^71^ which impairs cholinergic projections to the hippocampus. One approach to the symptomatic treatment of AD is to enhance cholinergic neurotransmission impaired during AD by blocking the enzymatic reaction by acetylcholinesterase, which is responsible for the breakdown of acetylcholine. In fact, cognitive decline can be improved by a group of drugs known as acetylcholinesterase inhibitors, such as donepezil.^72^^,^^73^

In most AD animal models, LTP deficits are observed that vary among different AD models, and although the reports on LTD are limited, they indicate enhanced induction of LTD.^66^ These observations seem to be consistent with the hypothesis that conditions that promote LTD might lead to loss of synapses, while conditions that promote LTP counteract LTD, preserving synaptic plasticity and brain connectivity.^66^^,^^74^ Thus, it can be useful to understand the predominant factors resulting in alterations of LTP and LTD observed in AD animal models for the development of AD therapy targeting synaptic plasticity. An increase in Aβ levels causes a decrease in the number of surface and synaptic AMPARs, consequently reducing dendritic spine density in hippocampal pyramidal neurons.^75^ Thus, we examined how the reduction in the number of AMPARs due to varying gene expression levels in dendritic spines affected the induction of LTP and LTD. Specifically, for the case in which only the initial input number of AMPARs consisted of GluA1/GluA2 heterotetramers and was reduced by half while retaining the other model parameters (called AD model), the influence on M1 mAChR-dependent and NMDAR-dependent LTP and LTD was examined (Table S4 in the SI table). The models of Ca^2+^ influx into the spine cytosol that were used in our previous study^21^ were employed (see STAR Methods) for NMDAR-dependent LTP and LTD simulations (Figure 2A).

**Figure 8:**
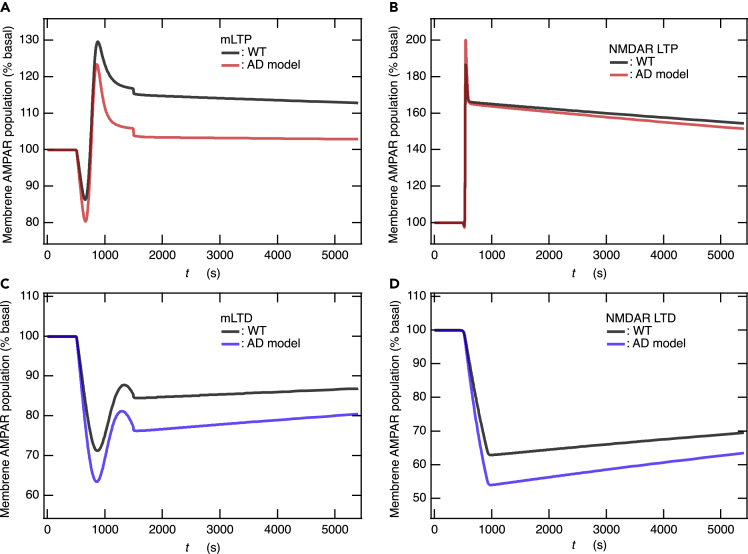
Reduction in AMPAR expression on the dendritic spine causes both reduced LTP and enhanced LTD (A and C) Time courses of the membrane AMPAR population for the M1 mAChR-dependent LTP (A) and LTD (C). (B and D) Time courses of the membrane AMPAR population for the NMDAR-dependent LTP (B) and LTD (D). The number of AMPARs was reduced by half in the AD model simulations but not in WT simulations.

In the basal level provided by steady-state simulation, the fraction of synaptic membrane AMPARs to the total AMPARs was 0.499 and 0.454, respectively, before and after the reduction in total AMPARs by half. Thus, AMPARs were almost equally lost from the membranes and the cytosol upon halving the total number of AMPARs. Surprisingly, strengthened induction of LTD was observed in both simulations with the M1 mAChR and NMDAR models (Figures 8C and 8D). In contrast, a reduced induction of LTP was observed for M1 mAChR (Figure 8A), while only a small reduction was observed for NMDAR (Figure 8B). Nevertheless, it is remarkable that AMPAR expression level influences both LTP and LTD, regardless of the mediators of synaptic plasticity. To investigate the reasons resulting in this synaptic dysfunction, we compared total fluxes of exocytosis, endocytosis, and myosin V_b_-mediated transport between the WT and the AD model during the inductions of mLTP and mLTD (see Figures 5 and S5 in the SI). Qualitatively, these fluxes during the induction of mLTP and mLTD in the AD model seem to be reduced to about half of those seen in the WT model, while the time courses of both the fluxes were very similar to the WT ones. Quantitatively, however, the cause behind the reduction of mLTP induction and the increasing amplitude of mLTD should be attributed to a slightly larger decrease in the total exocytosis flux compared to the total endocytosis flux. However, elucidation of the detailed mechanism is a future task.

Aging of the human brain causes cognitive decline in the elderly and is a major risk factor for AD. In fact, several neurotransmitter receptors that are centrally involved in synaptic plasticity, including GluA1 (encoded by *GRIA1*), show significantly reduced expression after the age of 40 years.^76^ Taken together, these observations suggest that either upregulation of neurotransmitter receptor genes or suppression of the downregulation could improve synaptic dysfunction during AD. Interestingly, calorie restriction effectively suppresses changes in age-dependent hippocampal gene expression, including *GRIA1*, suggesting its effectiveness in preventing AD.^77^

### Limitations of the study

In the current model, signaling between cholinergic, glutamatergic, and adrenergic Gq-coupled G protein-coupled receptors (GPCRs), namely, mAChRs, mGluRs, aARs, cannot be distinguished. The activation signals of mAChRs,^10^^,^^15^ mGluRs,^78^^,^^79^ and aARs^80^^,^^81^ partly converge on downstream signaling that causes IP3-mediated Ca^2+^ release from intracellular storage. Therefore, the results and conclusions derived from the present study are not necessarily intrinsic for mAChRs but can possibly be extended to Gq-coupled GPCRs. Remained work in the future is to examine whether the AMPAR trafficking network model can reproduce synaptic plasticity mediated by the other Gq-coupled GPCRs.

In the presented models, parameter changes other than the number of AMPARs caused by gene expression were not taken into consideration. Thus, the alterations in synaptic plasticity observed in AD, especially for NMDAR-dependent LTP, could be attributed to the influence of the expression of other genes, and further experimental and theoretical studies to identify them are necessary.

## STAR★Methods

### Key resources table

**Table:** 

REAGENT or RESOURCE	SOURCE	IDENTIFIER
**Software and algorithms**		

COPASI biochemical system simulator (v. 4.37)	COPASI	https://copasi.org/
Igol Pro (v. 8.04)	WaveMetrics	https://www.wavemetrics.com/

### Resource availability

#### Lead contact

The COPASI input data used to generate the data for the current study are available from the lead contact Tomonari Sumi (sumi@okayama-u.ac.jp) upon reasonable request.

#### Materials availability

This study did not generate new materials or reagents.

### Method details

#### Mathematical model

##### M1 mAChR-dependent Ca^2+^ dynamics (Figure 2B)

The flux of IP3 production mediated by phospholipase C (PLC) upon activation of M1 mAChR is used as the input of simulations for M1 mAChR-dependent LTP (mLTP) and LTD (mLTD). The concentration [IP3] is given by the following ordinary differential equation:

(S1) $$\frac{d\left[{IP}_{3}\right]}{dt}=V_{PLC}\left(t\right)\frac{{\left[{Ca}^{2+}\right]}^{2}}{{K_{PLC}}^{2}+{\left[{Ca}^{2+}\right]}^{2}}-k_{\deg}\left[{IP}_{3}\right]\text{,}$$

where the function $V_{PLC}\left(t\right)$ depends on agonist-induced activation of M1 mAChR and is modeled as

(S2) $$V_{PLC}\left(t\right)=V_{PLC}\left[\frac{1}{1+e^{-a_{sig}\left(t-t_{0}\right)}}-\frac{1}{1+e^{-a_{sig}\left(t-t_{1}\right)}}\right]{\left(\frac{t}{t_{0}}\right)}^{\alpha }\text{.}$$

$t_{0}$ and $t_{1}$ are the time of agonist application and the ending time, respectively. The parameter α is used to vary the production rate of IP3. In Equation 1, $\left[{Ca}^{2+}\right]$ is Ca^2+^ concentration in spine cytosol, $K_{PLC}$ is Ca^2+^ sensitivity of PLC,^26^ and $k_{\deg}$ is degradation rate of IP3. Ca^2+^ influx from ER into the spine cytosol is given as the sum of Ca^2+^ release flux through IP3 receptor from ER toward the spine cytosol and Ca^2+^ leak flux from ER into the spine cytosol,

(S3) $$J_{rel}^{ER}=\left\{k_{{IP}_{3}R}\left[{IP}_{3}\right]+k_{leak}\right\}\left({\left[{Ca}^{2+}\right]}_{ER}-\left[{Ca}^{2+}\right]\right)\text{,}$$

where ${\left[{Ca}^{2+}\right]}_{ER}$ is Ca^2+^ concentration inside ER, $k_{{IP}_{3}R}$ is the rate of Ca^2+^ release through IP3 receptor, and $k_{leak}$ is the rate of Ca^2+^ leak. Sarcoendoplasmic reticulum Ca^2+^–ATPases (SERCA) works in ER as an ATP-dependent calcium pump, and causes Ca^2+^ outflux from the spine cytosol into ER, which is given as

(S4) $$J_{SERCA}^{ER}=V_{SERCA}\frac{{\left[{Ca}^{2+}\right]}^{2}}{{K_{SERCA}}^{2}+{\left[{Ca}^{2+}\right]}^{2}}\text{,}$$

where $K_{SERCA}$ is Ca^2+^ sensitivity of SERCA.^25^ The total Ca^2+^ influx from the ER to the spine cytosol is given by

(S5) $$J_{in\text{-}out}^{ER}=J_{rel}^{ER}-J_{SERCA}^{ER}\text{.}$$

The concentration ${\left[{Ca}^{2+}\right]}_{ER}$ is given by the following ordinary differential equation:

(S6) $$\frac{d{\left[Ca^{2+}\right]}_{ER}}{dt}=-J_{in\text{-}out}^{ER}/\beta \text{,}$$

where β is the ratio of effective spine cytosol volume to effective ER volume. We used the parameter values shown in the table below for mLTP and mLTD stimulation.

**Table:** 

Parameters	mLTP	mLTD
$K_{PLC}$	0.4μM^83^	0.4μM^83^
α	0.4	0.9
$V_{PLC}$	$320\;\mu Ms^{-1}$	$320\;\mu Ms^{-1}$
$k_{\deg}$	$10\;s^{-1}$	$10\;s^{-1}$
$a_{sig}$	0.5 $s^{-1}$	0.5 $s^{-1}$
$k_{leak}$	$0.002\;s^{-1}$^83^	$0.002\;s^{-1}$^83^
$k_{{IP}_{3}R}$	$0.17\;\mu Ms^{-1}$	$0.17\;\mu Ms^{-1}$
$K_{SERCA}$	0.1μM^26^	0.1μM^26^
$V_{SERCA}$	$1.5\;\mu Ms^{-1}$	$1.5\;\mu Ms^{-1}$
β	$5.0\times 10^{5}$	$5.0\times 10^{5}$

##### NMDAR-dependent Ca^2+^ dynamics (Figure 2A)

Ca^2+^ pulses are used to induce NMDAR-dependent LTP and LTD as the input of simulations. A model of Ca^2+^ influx is responsible for regulation of [Ca^2+^], thus plays an important role in the network model of NMDAR-dependent LTP and LTD. Ca^2+^ influx into spine cytosol is used to elevate [Ca^2+^] and is modeled with two sigmoid functions:

$$J_{in}^{NMDAR}\left(t\right)=A_{sigmoid}\left\{\frac{1}{1+\exp\left[-a\left(t-t_{s}\right)\right]}-\frac{1}{1+\exp\left[-a\left(t-t_{f}\right)\right]}\right\}$$

We used the parameters shown in the table below for LTP and LTD stimulation.^21^

**Table:** 

LTP stimulation	Parameter values
$A_{sigmoid}$	1110 μmol/L
a	0.1 s^−1^
$t_{s}$	500 s
$t_{f}$	600 s
LTD stimulation	
$A_{sigmoid}$	820 μmol/L
a	0.1 s^−1^
$t_{s}$	500 s
$t_{f}$	950 s

The following explanations on the AMPAR trafficking model are same as that provided in our previous work on NMDAR-dependent LTP and LTD.^21^

##### The other Ca^2+^ dynamics

The model of Ca^2+^ extrusion used in this study (see below) is based on previous works.^40^^,^^84^ We also introduced a constant flow of Ca^2+^ into the cytosol as a zero order reaction, which counteracts the extrusion of Ca^2+^ from the cytosol, so that the system sustain a basal Ca^2+^ concentration of ∼50 nmol/L.^40^ This works in the total Ca^2+^ dynamics model together with either M1 mAChR-dependent Ca^2+^ dynamics model or NMDAR-dependent Ca^2+^ dynamics model.

Ca+PMCA=CaPMCA

CaPMCA→PMCA

Ca+NCX=CaNCX

CaNCX→NCX

Ca+SERCA=CaSERCA

$$Ca+CaSERCA={Ca}_{2}SERCA$$

$${Ca}_{2}SERCA=SERCA$$

$$Ca\_leak\_into\to Ca^{21}$$

#### cAMP synthesis from ATP

cAMP is synthesized by calcium-calmodulin bound adenylyl cyclase 1 (AC1). The related model used in this study (see below) is based on previous works.^85^^,^^86^

$$CaM+2Ca=CaM{Ca}_{2}$$

$$CaM{Ca}_{2}+2Ca=CaM{Ca}_{4}$$

$$AC1+CaMCa_{4}=AC1-CaMCa_{4}$$

$$AC1-CaMCa_{4}+ATP=AC1-CaMCa_{4}-ATP$$

$$AC1-CaMCa_{4}-ATP\to AC1-CaMCa_{4}+cAMP$$

#### cAMP degradation into ATP

cAMP is degraded through multiple pathways by phosphodiesterase type 1 (PDE1),^87^^,^^88^ phosphodiesterase type 4 (PDE4),^89^ and phosphorylated PDE4 (pPDE4) by PKA.^90^ The models for these cAMP degradation reactions used in this study (shown below) are based on previous works.^85^^,^^86^

$$PDE1+CaMCa_{4}=PDE1-CaMCa_{4}$$

$$PDE1-CaMCa_{4}+cAMP=PDE1-CaMCa_{4}-cAMP$$

$$PDE1-CaMCa_{4}-cAMP\to PDE1-CaMCa_{4}+AMP$$

AMP→ATP

PDE4+cAMP=PDE4−cAMP

PDE4−cAMP→PDE4+AMP

PDE4+PKAc=PDE4−PKAc

PDE4−PKAc→pPDE4+PKAc

pPDE4+cAMP=pPDE4−cAMP

pPDE4−cAMP→pPDE4+AMP

PDE4−cAMP+PKAc→PDE4−cAMP−PKAc

PDE4−cAMP−PKAc→PDE4−cAMP+PKAc

pPDE4→PDE4

#### Phosphorylation of the S845 of GluA1 by cAMP-bound PKA

PKA binds to A-kinase anchoring protein 150 (AKAP150)^29^^,^^32^^,^^33^ and these form a signaling complex at the postsynaptic plasma membrane. PKA is activated by cAMP and the resulting PKA, namely, PKAc, phosphorylates the S845 of GluA1 at the postsynaptic membrane.^28^^,^^31^ The model on the activation of PKA used in this study (shown below) is based on a previous work.^85^ The model of the phosphorylation of GluA1 S845 used in this study (shown below) is also according to previous works.^91^^,^^92^

PKA +2 cAMP = PKAcAMP_2_

PKAcAMP_2_ + 2 cAMP = PKAcAMP_4_

PKAcAMP_4_ = R2_cAMP_4_ + 2 PKAc

R2_cAMP_4_ = R2 + 4 cAMP (The parameter obtained from^93^)

R2 + 2 PKAc → PKA (The parameters are adjusted in^21^)

S845-GluA1 + PKAc = GluA1-PKAc

S845-GluA1-PKAc → pS845-GluA1 + PKAc

##### Dephosphorylation of the S845 of GluA1 by Ca^2+^-calmodulin-bound PP2B

Protein phosphatase 2B (PP2B, also known as Calcineurin) binds to AKAP150^29^^,^^32^^,^^33^ and forms a signaling complex with AKAP150 at the postsynaptic plasma membrane. Calmodulin (CaM) has Ca^2+^-binding affinity and PP2B is activated by calcium-bound CaM.^94^ The calcium-calmodulin-bound PP2B dephosphorylates the serine 845 of GluA1 at the postsynaptic plasma membrane.^30^ The model of Ca^2+^-dependent activation of PP2B used in this study (shown below) is provided by previous works.^92^^,^^94^^,^^95^ The model on the dephosphorylation of serine 845 GluA1 used in this study (shown below) is also presented by previous works.^91^^,^^92^

CaM +2 Ca = CaMCa_2_

CaMCa_2_ + 2 Ca = CaMCa_4_

PP2B + CaM = PP2B-CaM

PP2B + CaMCa_2_ = PP2B-CaMCa_2_

PP2B + CaMCa_4_ = PP2B-CaMCa_4_

PP2B-CaM +2 Ca = PP2B-CaMCa_2_

PP2B-CaMCa_2_ + 2 Ca = PP2B-CaMCa_4_

pS845-GluA1 + PP2B-CaMCa_4_ = pS845-GluA1-PP2B-CaMCa_4_

pS845-GluA1-PP2B-CaMCa_4_→ GluA1 + PP2B-CaMCa_4_

#### Phosphorylation of the S880 of GluA2 by Ca^2+^-bound PKC

Protein kinase C (PKC) also forms a signaling complex with the AKAP150 ^29^ at the postsynaptic plasma membrane. PKC has three C2 domains that bind Ca^2+^ ions,^96^ and Ca^2+^-bound PKC phosphorylates the S880 site of GluA2 at the postsynaptic membrane.^34^ The model of Ca^2+^-binding to PKC used in this study (shown below) is provided by previous works.^40^ The model of the phosphorylation of the S880 of GluA2 by Ca^2+^-bound PKC is according to a previous work^97^

PKC + Ca = CaPKC

CaPKC + Ca = Ca_2_PKC

Ca_2_PKC + Ca = Ca_3_PKC

S880-GluA2 + Ca_3_PKC = S880-GluA2-Ca_3_PKC

S880-GluA2-Ca_3_PKC → pS880-GluA2 + Ca_3_PKC

#### PICK1 that triggers endocytosis of AMPARs

Protein interacting with C-kinase 1 (PICK1) is a Ca^2+^-sensor protein that has two Ca^2+^-binding sites and Ca^2+^-bound PICK1 triggers endocytosis of AMPARs by binding to phosphorylated S880 of GluA2.^34^^,^^37^^,^^38^ The Ca^2+^-binding constants, *K*_1b_ and *K*_2b_, have been experimentally determined to be 1.80 and 0.27 L/μmol, respectively.^98^ The rate constants for the forward and backward reaction shown below can be modeled using *K*_1b_ and *K*_2b_.

PICK1 + Ca = CaPICK1 k_1f_⇄ k_1ba_

CaPICK1 + Ca = Ca_2_PICK1 k_2f_⇄ k_2b_

If we assumed the forward reaction constants, *k*_1f_ and *k*_2f_, to be 21.6 and 10.8 L/(μmol s),^21^ respectively, the backward reaction constants, *k*_1b_ and *k*_2b_, were determined to be 12 and 40 s^−1^, respectively, by using *k*_1b_ = *k*_1f_/*K*_1b_ and *k*_2b_ = *k*_2f_/*K*_2b_. These forward reaction constants were adjusted so that NMDAR-dependent inductions of LTP and LTD were reproduced by the AMPAR trafficking model with these parameters.^21^

#### Syt1 that triggers exocytosis of AMPARs

Synaptic vesicle protein, synaptotagmin 1 (Syt1) is a Ca^2+^-sensor protein that has two Ca^2+^-binding domains, C2A and C2B.^47^ The Ca^2+^-bound Syt1 triggers exocytosis of AMPARs by cooperating with synaptic vesicle protein synaptobrevin-2/VAMP2, synaptic membrane protein synaptotagmin 7 (Syt7), and amongst others.^48^^,^^49^^,^^50^^,^^51^^,^^53^ In fact, it has been observed that Ca^2+^-binding site mutations of Syt1 in both the C2A and C2B domains block hippocampal LTP.^51^ Therefore, it would be appropriate to use Ca^2+^-binding constants of Syt1 to model a Ca^2+^-dependent regulating factor on the exocytosis mediated by Syt1 together with Syt7, synaptobrevin-2/VAMP2, and complexin, amongst others.^47^^,^^48^^,^^49^^,^^50^^,^^51^^,^^52^ The C2A and C2B domain of Syt1 bind three and two Ca^2+^ ions, respectively. Ca^2+^-binding constants for the C2A domain, *K*_A1_, *K*_A2_, and *K*_A3_, have been experimentally determined to be 2.04 x 10^−2^, 2.04 x 10^−3^, and 3.21 x 10^−4^ L/μmol, respectively, and that for the C2B domain, *K*_B1_, has been determined to be 7.04 x 10^−3^ L/μmol.^47^ The reaction equations and each forward and backward rate constant are defined as follows:

Syt1 + Ca = (Ca)_A_Syt1 k_A1f_⇄ k_A1b_

(Ca)_A_Syt1 + Ca = (Ca_2_)_A_Syt1 k_A2f_⇄ k_A2b_

(Ca_2_)_A_Syt1 + Ca = (Ca_2_)_A_(Ca)_B_Syt1 k_B3f_⇄ k_B3b_

(Ca_2_)_A_(Ca)_B_Syt1 + Ca = (Ca_2_)_A_(Ca_2_)_B_Syt1 k_B4f_⇄ k_B4b_

(Ca_2_)_A_(Ca_2_)_B_Syt1 + Ca = (Ca_3_)_A_(Ca_2_)_B_Syt1 k_A5f_⇄ k_A5b_

If we assumed these forward rate constants, *k*_A1f_, *k*_A2f_, *k*_B3f_, *k*_B4f_, and *k*_A5f_, to be 2.0, 1.0, 1.0, 1.0, and 0.5 L/(μmol s),^21^ respectively, those backward rate constants, *k*_A1b_, *k*_A2b_, *k*_B3b_, *k*_B4b_, and *k*_A5b_, were determined to be 97, 488, 142, 142, and 1560 s^−1^, respectively, by using the experimentally determined binding constants, *K*_A1_, *K*_A2_, *K*_A3_, and *K*_B1_. These forward reaction constants were adjusted so that NMDAR-dependent inductions of LTP and LTD were reproduced by the AMPAR trafficking model with these parameters.^21^

#### Network model on phosphorylation/dephosphorylation dynamics of AMPARs at the synaptic membrane

We incorporated GluA1/A2 heterotetramer, which is the most dominant subtype at hippocampal neurons,^27^ as the AMPAR model. The state of the AMPAR is identified by the 2 x 2 matrix shown in Figure S6. The upper left and right element indicate the numbers of phosphorylated S845 of GluA1 and of phosphorylated S880 of GluA2, respectively. The lower left and right element indicate the number of synaptic associated protein 97 kDa (SAP97)^32^ bound to phosphorylated GluA1 and the number of PICK1 bound to phosphorylated GluA2, respectively. If the number of lower right element is 0, 1, and 2, the number of glutamate receptor interacting protein 1 (GRIP1)^32^^,^^36^ bound to dephosphorylated GluA2 corresponds to 2, 1, and 0, respectively. State transitions occur between states of neighbor phosphorylation levels under keeping the number of AMPAR interaction proteins and between neighbor states inside the same phosphorylation level. The state of AMPARs that are incorporated into the postsynaptic membrane by Sty1-mediated exocytosis corresponds to the state with the phosphorylated levels of both GluA1 and GluA2 being zero and without SAP97 but with GRIP1 (the bottom left panel in Figure S6). The endocytosis of AMPARs that is mediated by PICK1 occurs through three states at the phosphorylated level of the bottom right (Figure S6).

#### Network model on the dynamics of PICK1-binding to AMPARs and of Ca^2+^-binding to PICK1-bond AMPARs

In addition to PICK1 binding to AMPARs, which is already considered in Figure S6, we also take into consideration CaPICK1-binding and Ca_2_PICK1-binding to the AMPARs with two dephosphorylated GluA1s and two phosphorylated GluA2s (i.e., the AMPARs with three states at the phosphorylated level of the bottom right in Figure S7), and also take into consideration Ca^2+^-binding to the PICK1-bound AMPARs (Figure S7). Three examples showing the correspondences between the 2 x 2 and 1 x 3 matrix representations are provided at the top of Figure S7. All the possible state transitions for Ca^2+^-binding and PICK1-binding due to the states listed at the left-hand side of Figure S7 are given as horizontal transitions in Figure S7. To satisfy the detailed balance condition, we assume all the rates of PICK1-, CaPICK1-, and Ca_2_PICK1-binding to AMPAR to be equivalent and also assume all the rates of PICK1-, CaPICK1-, and Ca_2_PICK1-dissociation from AMPAR to be equivalent.

#### Network model on dephosphorylation dynamics of GluA2 that regulates PICK1 dissociation from endocytic AMPARs and GRIP1-binding to those in the cytosol

Three kinds of one PICK1-bound AMPAR and six kinds of two PICK1-bound AMPAR, which appear at Figure S7, are internalized as recycling endosomes by PICK1-mediated endocytosis (these are indicated with the blue and red box on the bottom left of Figure S8). We assume that the serine 880 of GluA2 of internalized AMPARs is dephosphorylated on the recycling endosomes by protein phosphatase 2A (PP2A).^97^ PICK1s that bind to the GluA2 are dissociated and instead GRIP1 binds to the dephosphorylated S880 of GluA2.^32^^,^^36^ In the present study, we assume that the recycling endosomes containing the AMPARs with the state at the upper right of Figure S8 are transported by molecular motor myosin V_b_ toward the postsynaptic membrane.^41^^,^^42^^,^^43^

#### An approximate treatment on the relation between recycling endosome and Syt1

Ca^2+^-sensor synaptic vesicle protein synaptotagmin 1 (Syt1) mediates exocytosis of AMPARs, and the AMPARs are internalized into the cytosol as recycling endosomes. The recycling endosomes containing AMPARs undergo diffusion in the cytosol and those bound to molecular motor myosin V_b_ are transported to the peri-synaptic and synaptic membrane surface. On the basis of an analogy with Syt1-mediated synaptic vesicle exocytosis at peri-synaptic and synaptic membrane that results in neurotransmitter release,^48^^,^^49^^,^^50^^,^^51^^,^^53^ the Syt1 proteins are expected to be carried by the endocytic vesicles along with AMPARs in postsynaptic neurons as well. However, the network model becomes extremely complicated in the cytosol, if multiple Ca^2+^-binding Syt1 species are included in the recycling endosomes together with AMPARs with various states. In the present work, for simplification, we introduce the following approximation: the Syt1 proteins are artificially remained at the postsynaptic membrane when AMPARs are internalized as the recycling endosomes; the Syt1 proteins left at the postsynaptic membrane bind to the recycling endosomes as immediately as myosin-V_b_ molecules transport the recycling endosomes to peri-synaptic and synaptic membrane surface.

## Data Availability

• Data reported in this paper will be shared by the lead contact upon request. • The COPASI input data used to generate the data for the current study are available from the lead contact upon reasonable request. This paper does not report original code. • Any additional information required to reanalyze the data reported in this paper is available from the lead contact upon request.

## Supplemental information

Document S1. Figure S1-S8 and Table S1, S3-S6

Table S2. Reactions and parameters of the network model on hippocampal bidirectional synaptic plasticity, related to STAR Methods
