# Supplementary material for: Muscarinic acetylcholine receptor-dependent and NMDA receptor-dependent LTP and LTD share the common AMPAR trafficking pathway
Source: iScience. 2023 Feb 3;26(3):106133. doi: 10.1016/j.isci.2023.106133 (PMC9972575; doi:10.1016/j.isci.2023.106133)
Supplement: Document S1. Figure S1-S8 and Table S1, S3-S6 [file mmc1.pdf]

iScience, Volume *26*

Supplemental information

Muscarinic acetylcholine receptor-dependent and NMDA receptor-dependent LTP and LTD share the common AMPAR trafficking pathway

Tomonari Sumi and Kouji Harada

SUPPLEMENTAL INFORMATION FIGURES

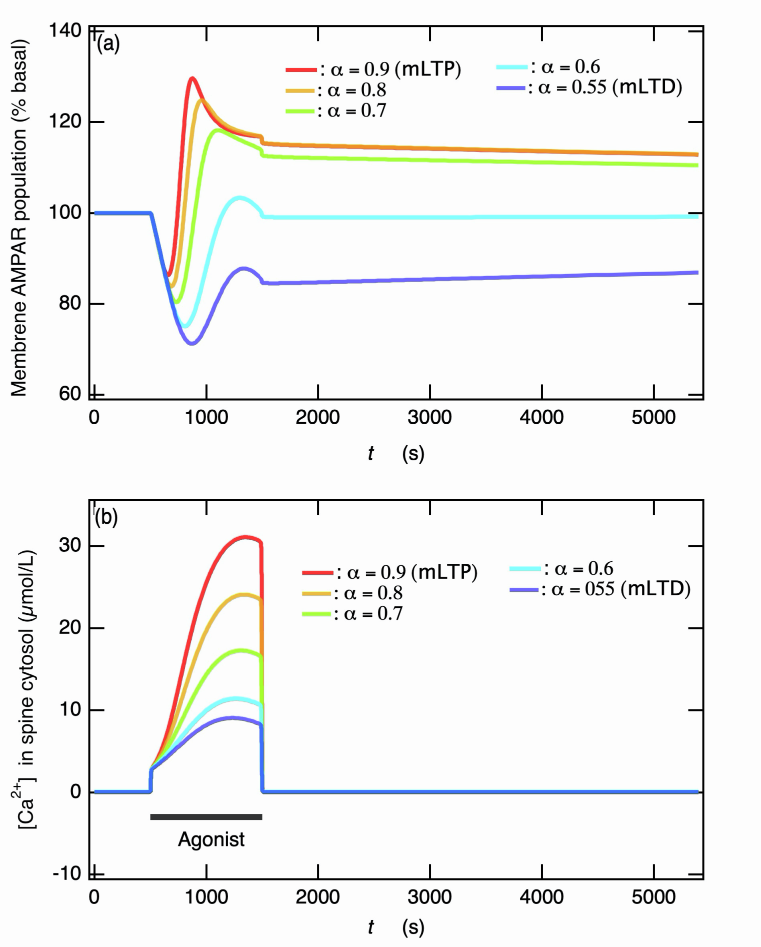

Figure S1. Population of membrane AMPARs monotonically varies from the mLTD to mLTP induction upon gradually increasing [IP3] and resulting raise in cytosolic [Ca^{2+}], related to the information for Fig. 4. Increasing the parameter α on PCL-mediated IP3 production increases Ca^{2+} influx from ER into spine cytosol. The results for $\alpha = 0.55$ and 0.9 correspond to the induction of mLTD and mLTP depicted in Fig. 4a. The time course of (a) membrane AMPAR population and (b) cytosolic [Ca^{2+}] upon varying the parameter α. The duration of M1 mAChR activation is 1000 s.

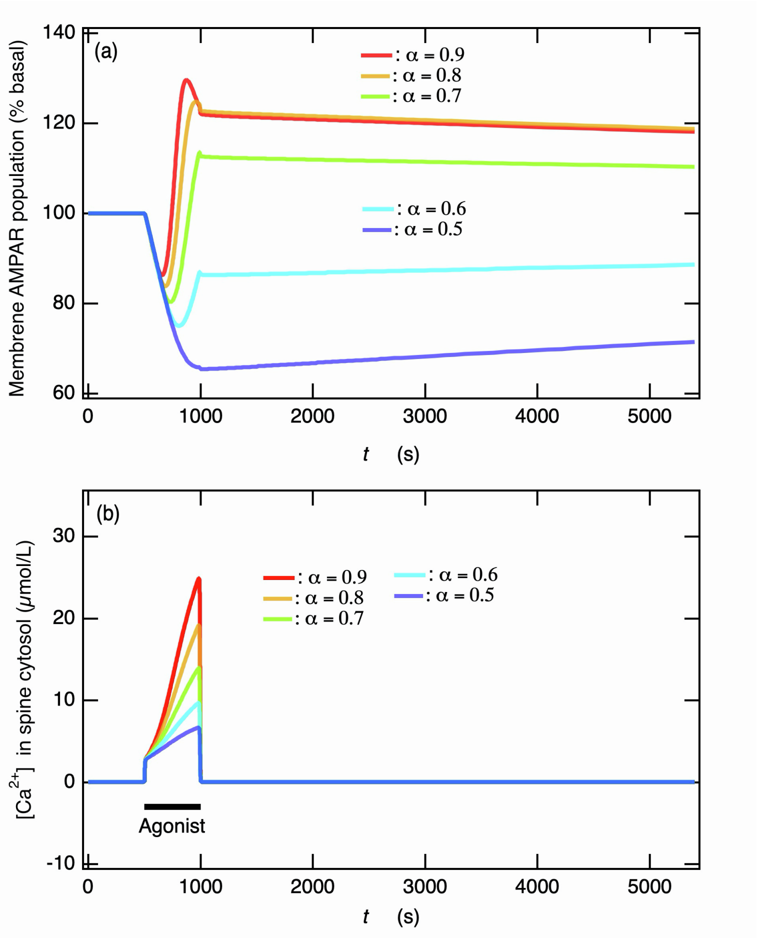

Figure S2. Qualitatively similar mLTP induction (to Fig. 4) is observed even when the duration of M1 mAChR activation is reduced by half, related to the information for Fig. 4. (a) and (b) are same as that shown in Fig. S1, whereas the duration of M1 mAChR activation is the half.

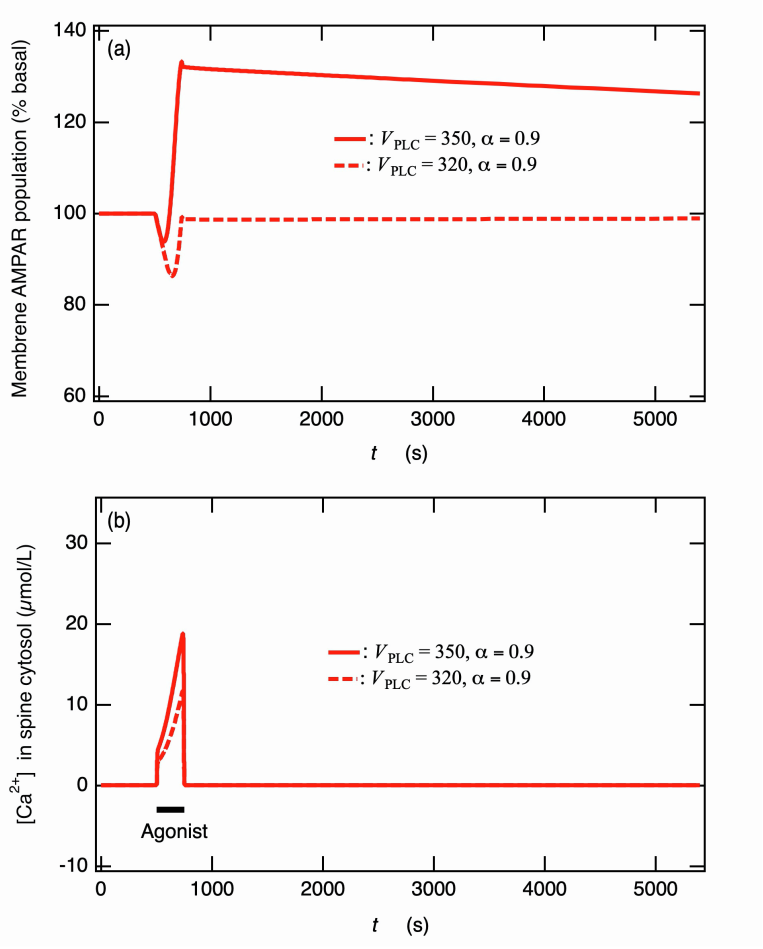

Figure S3. Shorter activation duration decreases the initial reduction in membrane AMPAR population compared with Fig. 4a, related to the information for Fig. 4. The time course of (a) membrane AMPAR population and (b) cytosolic [Ca^{2+}]. The duration of M1 mAChR activation is 250 s. The induction of mLTP was recovered by an increase in IP3 production rate V_{PLC} (Eq. S2), even if the activation duration was further decreased.

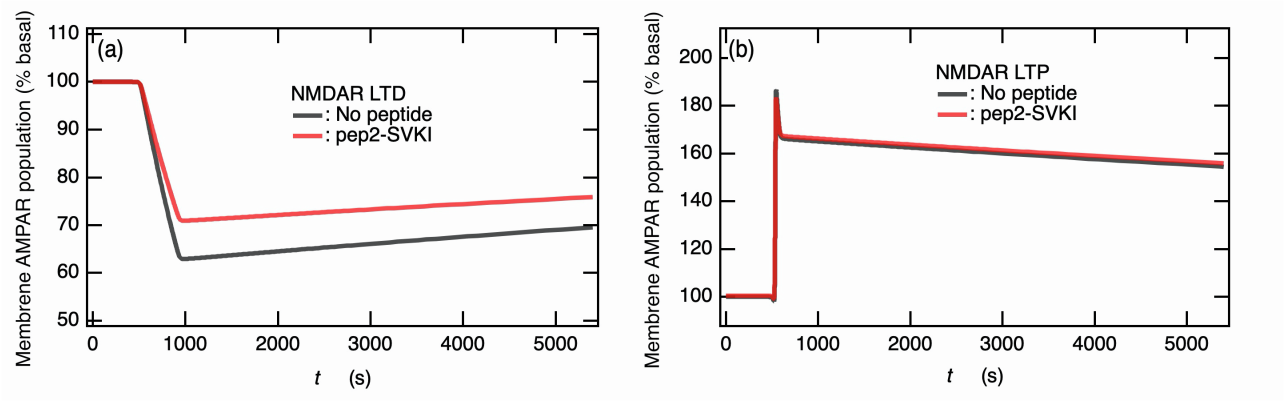

Figure S4. The interference of interactions between GluA2 and PICK1 by pep2-SVKI impairs NMDAR-dependent LTD, while does not affect NMDAR-dependent LTP at all, related to the information for Fig. 6. (a) LTD (b) LTP. These simulation results are consistent with experimental observation for adult PICK1-KO mice [1].

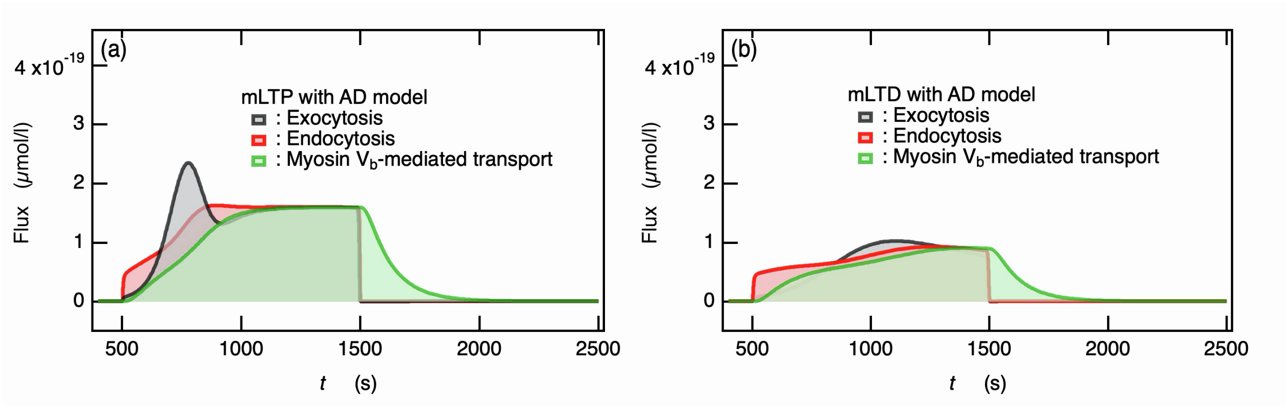

Figure S5. Varying balance between exocytosis and endocytosis yields reduced LTP and enhanced LTD in the AD model, related to the information for Figs. 8a and 8c. (a) Fluxes during induction of mLTP in the AD model. (b) Fluxes during induction of mLTD in the AD model. In the AD model, total AMPARs were reduced by half, as a result, the membrane AMPARs were decreased to be almost half at the basal level, indicating AMPARs were almost equally lost from the membranes and the cytosol.

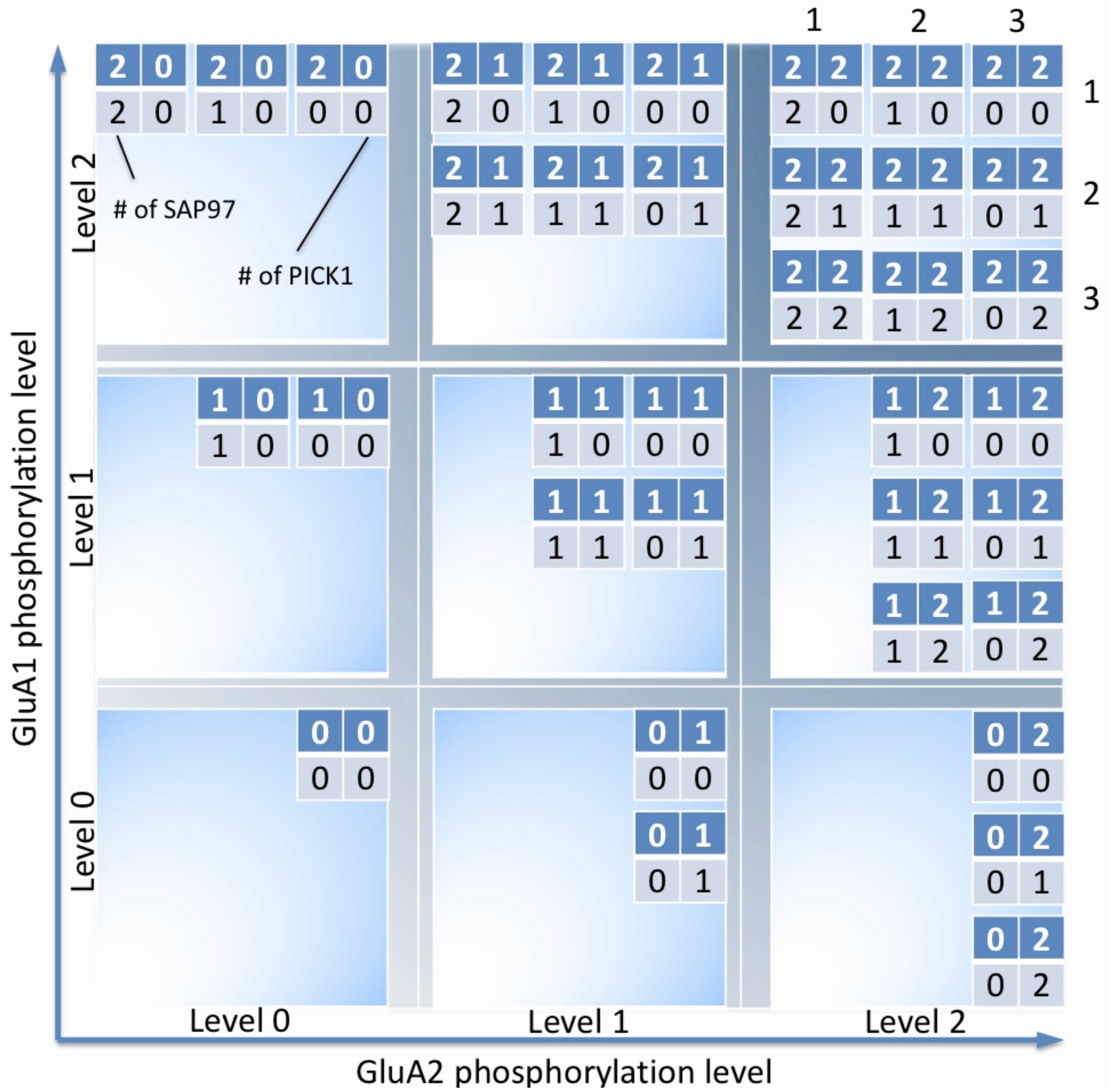

Figure S6. Network model on phosphorylation/dephosphorylation dynamics of AMPARs at the synaptic membrane, related to STAR Methods.

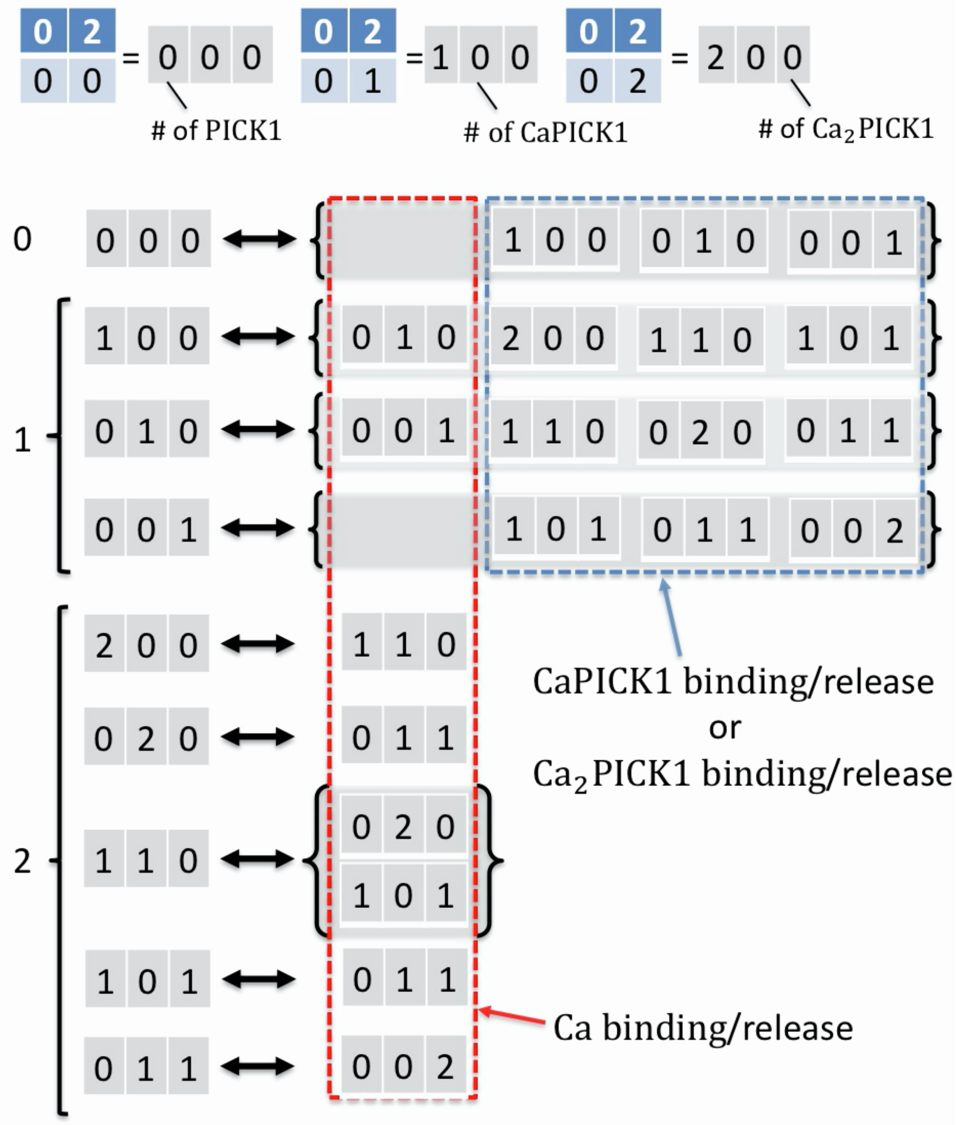

Figure S7. Network model on the dynamics of PICK1-binding to AMPARs and of Ca^{2+}-binding to PICK1-bond AMPARs, related to STAR Methods. Here, the AMPARs with two dephosphorylated GluA1 and two phosphorylated GluA2, namely, the AMPARs with three states at the phosphorylated level of the bottom right in Fig. S7, are taken into consideration. Three examples showing the correspondences between the 2 x 2 and 1 x 3 matrix representations are given at the top of Fig. S7.

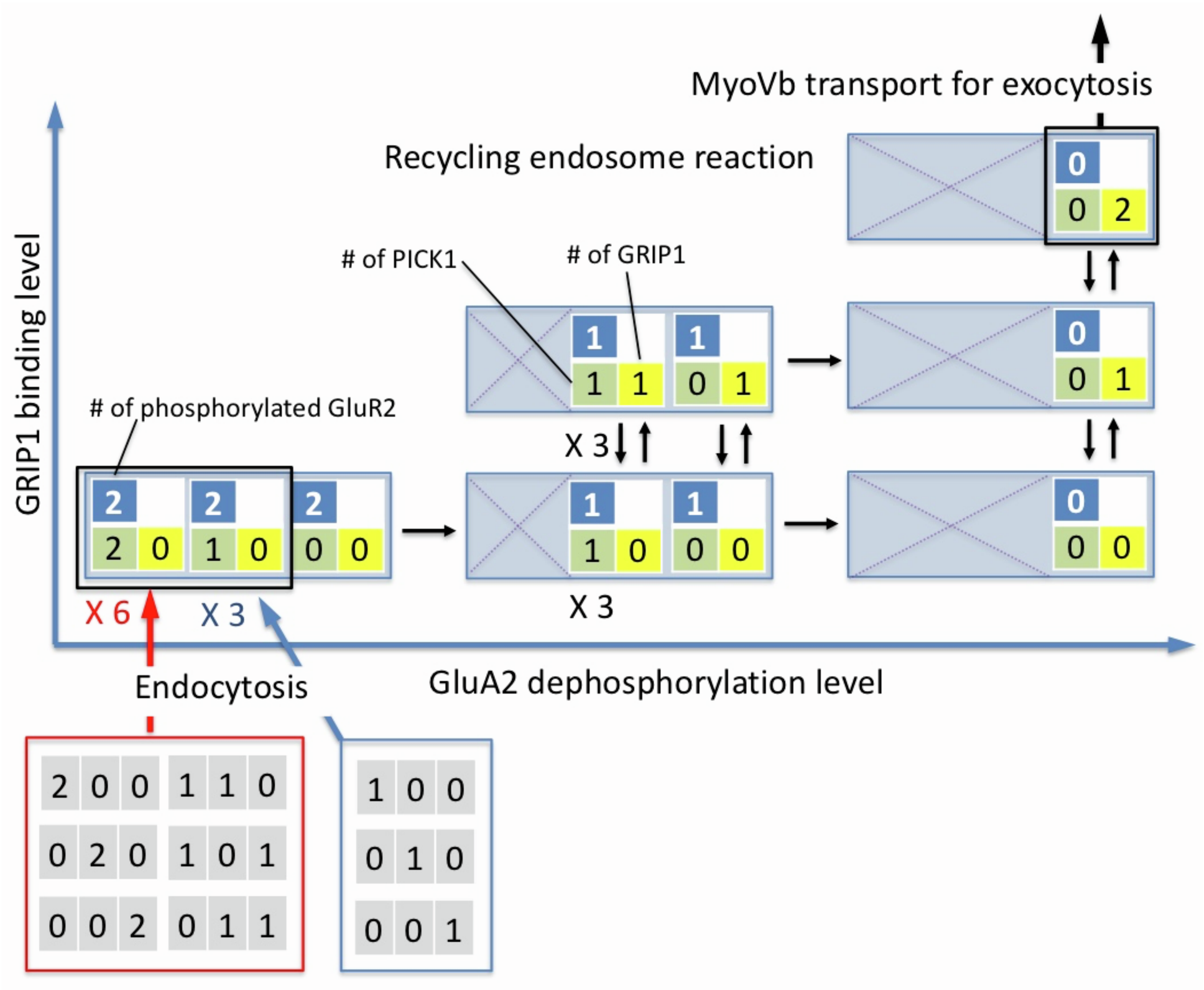

Figure S8. Network model on dephosphorylation dynamics of GluA2 that regulates PICK1-dissociation from endocytic AMPARs and GRIP1-binding to those in the cytosol, related to STAR Methods.

SUPPLEMENTAL INFORMATION TABLES

Table S1. Parameters of the network model at the initial condition, related to STAR Methods. For simplification of making input data, we set the initial concentrations for the other species that are not provided in this table and all the simulations are started from the steady state condition.

	**Volume (l)**	**Ref.**
Compartment size	6.00E-17	[2,3]
**Species**	**Initial concentration (μmol/l)**	
AC1	1.6686	[4]
AC1-CaMCa4	0.14	[5]
AC1-CaMCa4-ATP	0.1422	[4]
AMP	0.495	[4]
ATP	1998.64	[4]
Ca	0.05	[2]
CaM	8.775	[4]
CaMCa2	0.291	[4]
CaMCa4	0.01	[4]
cAMP	0.027	[4]
MyoV	10	[5]
NCX	0.111	[2]
PDE1	3.457	[4]
PDE1-CaMCa4	0.489	[4]
PDE1-CaMCa4-cAMP	0.001	[4]
PDE4	2.766	[4]
PDE4-cAMP	0.034	[4]
PDE4-PKAc	0.025	[4]
PICK1	1	[5]
PKA	1.7634	[4]
PKAcAMP2	0.3426	[4]
PKAcAMP4	0.0102	[4]
PKC	3	[5]

PMCA	0.277	[2]
PP2A	1.1	[5]
PP2B-CaM	2.99	[4]
PP2B-CaMCa$_2$	0.989	[4]
PP2B-CaMCa$_4$	0.1	[4]
pPDE4	0.122	[4]
pPDE4-cAMP	0.001	[4]
A1A2(GRIP$_2$)	2	[5]
R2_cAMP$_4$	0.0096	[5]
SAP	4	[5]
SERCA	1.66	[2]
Syt1	4	[5]

Table S3. Parameters for peptide pep2-SVKI inhibition on PICK1-mediated endocytosis, related to the information for Fig. 6. All the parameters of endocytosis mediated by Ca^{2+}-binding PICK1 was reduced to be 80 %.

No.	Reaction	k_f	k_b	Ref.
85	A1A2(pS$_2$-Ca$_2$PICK1) -> A1A2endo(pS$_2$-Ca$_2$PICK1)	0.0008 1/s		This work
86	A1A2(pS$_2$-Ca$_2$PICK1-Ca$_2$PICK1) -> A1A2endo(pS$_2$-Ca$_2$PICK1-Ca$_2$PICK1)	0.048 1/s		This work
87	A1A2(pS$_2$-CaPICK1) -> A1A2endo(pS$_2$-CaPICK1)	0.0004 1/s		This work
88	A1A2(pS$_2$-CaPICK1-Ca$_2$PICK1) -> A1A2endo(pS$_2$-CaPICK1-Ca$_2$PICK1)	0.016 1/s		This work
89	A1A2(pS$_2$-CaPICK1-CaPICK1) -> A1A2endo(pS$_2$-CaPICK1-CaPICK1)	0.0008 1/s		This work
91	A1A2(pS$_2$-PICK1-Ca$_2$PICK1) -> A1A2endo(pS$_2$-PICK1-Ca$_2$PICK1)	0.0008 1/s		This work
92	A1A2(pS$_2$-PICK1-CaPICK1) -> A1A2endo(pS$_2$-PICK1-CaPICK1)	0.00016 1/s		This work

Table S4. Comparison of parameters of the network model at the initial condition between the WT and AD models, related to the information for Fig. 8.

Species	Initial concentration for WT model (μmol/l)	Initial concentration for AD model (μmol/l)
A1A2(GRIP$_2$)	2	1
GRIP1	0	2

Table S5. Input of Ca^{2+} pulses for three times LFSs employed in an occlusion experiment of mLTD after saturated induction of NMDAR-dependent LTD [6], related to the information for Fig. 7. The mLTD stimulation, which is same as that used in Fig. 4, following three episodes of LFS is applied for 1000 s between 9800 s and 10800 s.

	LFS stimulation 1	LFS stimulation 2	LFS stimulation 3
$A_{sigmoid}$	920 μmol/l	920 μmol/l	920 μmol/l
a	0.1 s^{-1}	0.1 s^{-1}	0.1 s^{-1}
t_s	500 s	2600 s	5300 s
t_f	1400 s	3500 s	6200 s

Table S6. Input parameter changed in the simulation for the occlusion experiment of mLTD after saturated induction of NMDAR-dependent LTD [6], related to the information for Fig. 7.

Species	Initial concentration for AD model (μmol/l)
A1A2(GRIP$_2$)	6

REFERENCES

1. Volk, L., Kim, C.-H., Takamiya, K., Yu, Y. & Huganir, R. L. (2010) Developmental regulation of protein interacting with C kinase 1 (PICK1) function in hippocampal synaptic plasticity and learning. *PNAS* **107,** 21784–21789.
2. Antunes, G., Roque, A. C. & Simoes-de-Souza, F. M. (2016) Stochastic Induction of Long-Term Potentiation and Long-Term Depression. *Sci. Rep.* **6,** 30899–11.
3. HARRIS, K. M. & STEVENS, J. K. (1988) Dendritic Spines of Rat Cerebellar Purkinje-Cells - Serial Electron-Microscopy with Reference to Their Biophysical Characteristics. *J. Neurosci.* **8,** 4455–4469.
4. Chay, A., Zamparo, I., Koschinski, A., Zaccolo, M. & Blackwell, K. T. (2016) Control of βAR- and N-methyl-D-aspartate (NMDA) Receptor-Dependent cAMP Dynamics in Hippocampal Neurons. *PLoS Comput Biol* **12,** e1004735.
5. Sumi, T. & Harada, K. (2020) Mechanism underlying hippocampal long-term potentiation and depression based on competition between endocytosis and exocytosis of AMPA receptors. *Sci. Rep.* **10,** 14711–14.
6. Volk, L. J., Pfeiffer, B. E., Gibson, J. R. & Huber, K. M. (2007) Multiple Gq-coupled receptors converge on a common protein synthesis-dependent long-term depression that is affected in fragile X syndrome mental retardation. *J. Neurosci.* **27,** 11624–11634.
